# Probiotic modulation of gut microbiota in preterm infants: a narrative review of clinical evidence, mechanisms, and translational gaps

**DOI:** 10.3389/fmicb.2026.1945012

**Published:** 2026-10-29

**Authors:** Dongke Xie, Haokun Yuan, Deshuang Zhang

**Affiliations:** 1 Department of Pediatric Surgery, Children’s Medical Center, The Affiliated Hospital of Southwest Medical University, Luzhou, Sichuan, China; 2 Sichuan Clinical Research Center for Birth Defects, Luzhou, Sichuan, China; 3 Department of Neonatology, Children’s Medical Center, The Affiliated Hospital of Southwest Medical University, Luzhou, Sichuan, China

**Keywords:** clinical evidence, gut microbiota, microbiome modulation, preterm infants, probiotics, translational gaps

## Abstract

Preterm infants are at a heightened risk for gut dysbiosis, a condition characterized by reduced microbial diversity, delayed colonization by beneficial commensals such as *Bifidobacterium*, and overgrowth of opportunistic pathogens. This disrupted microbial ecosystem is intricately linked to the pathogenesis of life-threatening conditions, including necrotizing enterocolitis (NEC), late-onset sepsis (LOS), and feeding intolerance, and is also implicated in long-term neurodevelopmental and respiratory outcomes. Probiotic supplementation has emerged as a prominent strategy to shift the microbiota toward a more eubiotic-like composition and mitigate these risks. This narrative review synthesizes evidence from a wide range of randomized controlled trials, observational studies, systematic reviews, and meta-analyses to evaluate the clinical efficacy and microbiological impact of probiotics in preterm populations. The evidence indicates that probiotic interventions, particularly specific multi-strain formulations containing well-defined *Lactobacillus* and *Bifidobacterium* strains, are associated with significant reductions in the incidence of severe NEC (risk ratio 0.38–0.57) and all-cause mortality (risk ratio 0.69). However, their effect on preventing LOS and reducing colonization by multidrug-resistant organisms is less consistent. Probiotics consistently enhance the intestinal abundance of beneficial bacteria, modulate metabolic profiles, and reduce markers of intestinal inflammation. However, effects are strain-specific and cannot be extrapolated across products; future trials should report full strain identity, dose, viability, and product composition. Emerging evidence also suggests a potential role for probiotics in influencing immune cell development and the gut-brain axis, with implications for neurodevelopment and seizure prevention, though these findings are preliminary. This review also addresses critical challenges, including the considerable heterogeneity in study designs, probiotic strains, and clinical protocols, safety concerns in extremely low-birth-weight infants, and the methodological issues related to product quality and taxonomic identification. We conclude that multi-strain probiotics reduce severe NEC and all-cause mortality, but evidence for LOS is inconsistent and neurodevelopmental benefit remains unproven. Routine universal use is therefore not justified; translation requires product-specific trials and regulatory-grade quality control.

## 1 Introduction

Preterm birth, defined as delivery before 37 completed weeks of gestation, remains a leading cause of neonatal mortality and morbidity worldwide, with particularly high burdens in both high-income and low- and middle-income settings (Van Rossum et al., 2024; Wang et al., 2023; Pei et al., 2025). Infants born prematurely face a convergence of physiological, environmental, and iatrogenic challenges that profoundly disrupt postnatal development. Among the most consequential of these disruptions is the aberrant establishment of the gut microbiome—gut dysbiosis—which is increasingly recognized as a central mediator of neonatal morbidity (Xiang et al., 2023; Tao et al., 2026).

Preterm infants typically exhibit low microbial diversity, delayed colonization by commensal anaerobes, and overgrowth of opportunistic pathogens (Jia et al., 2022; Chen et al., 2023; Chen et al., 2024). This dysbiotic state has been associated with necrotizing enterocolitis (NEC), late-onset sepsis (LOS), feeding intolerance, bronchopulmonary dysplasia (BPD), and long-term neurodevelopmental deficits (Pei et al., 2025; Tao et al., 2026; Rozé et al., 2017; Zhang et al., 2022; Abrahamsson et al., 2026; Hutchinson et al., 2024). Given the central role of dysbiosis in driving adverse outcomes, interventions aimed at modulating the gut microbiota have garnered substantial scientific and clinical interest. Probiotics are defined by FAO/WHO as live microorganisms that, when administered in adequate amounts, confer a health benefit on the host (FAO/WHO, 2006). Importantly, this definition does not imply class-level effects: probiotic properties, including colonization capacity, metabolic activity, immune modulation, and clinical efficacy, are strain-specific. Findings obtained with one strain or formulation cannot be extrapolated to other strains, species, or genera (FAO/WHO, 2006; Hill et al., 2014). However, the evidence is not uniformly positive; some large trials have failed to demonstrate significant reductions in colonization by multidrug-resistant organisms (MDRO+) or improvements in sepsis rates, and the immunological consequences of probiotic-driven microbial shifts remain incompletely understood (Van Rossum et al., 2024; Juber et al., 2021; Smout et al., 2026).

Despite the promise of probiotics, significant gaps and controversies persist. The field is characterized by marked heterogeneity in study designs, probiotic strains and formulations, dosage regimens, and patient populations, complicating direct comparisons and the formulation of universal clinical guidelines (Thye et al., 2026; Lefèvre et al., 2024; Aceti et al., 2015). Because probiotic effects are strain-specific, we report strain designations whenever available and interpret evidence at the strain/formulation level; genus-level statements are used only as broad summaries and should not be interpreted as class effects (FAO/WHO, 2006; Hill et al., 2014). Concerns regarding the quality control and taxonomic accuracy of commercial probiotic products have also been raised, with studies revealing discrepancies between label claims and actual bacterial content (Lewis et al., 2016). Moreover, safety considerations, particularly in the most vulnerable extremely low birth weight (ELBW) infants, remain a subject of ongoing debate, as case reports of probiotic-associated sepsis, although rare, have tempered the enthusiasm for routine universal administration (Juber et al., 2021; Thye et al., 2026). Additionally, the mechanistic pathways through which probiotics exert their effects—whether via direct microbial competition, production of antimicrobial metabolites, modulation of host immunity, or interactions with the enteric nervous system—are only beginning to be elucidated (Rühle et al., 2024; Qin et al., 2026; Chen et al., 2026). The long-term impact of early probiotic exposure on neurodevelopment, metabolic health, and the acquisition of antibiotic resistance genes also requires further investigation (Upadhyay et al., 2020; Bargheet et al., 2023; Fernandes et al., 2024).

This narrative review aims to synthesize the current literature on probiotics for modulating the gut microbiota in preterm infants. We searched PubMed/MEDLINE, Embase, Web of Science, and the Cochrane Library (inception to March 2026) using terms related to preterm infants, gut microbiota, and probiotics; included randomized trials, observational studies, systematic reviews, meta-analyses, and selected preclinical studies; and excluded case reports, editorials, and non-peer-reviewed articles. Evidence was appraised narratively, with attention to study design, risk of bias, strain specificity, dose, and gestational age; no formal meta-analysis was performed. We evaluate clinical outcomes (NEC, sepsis, and others), microbiological and metabolic changes, mechanisms (including immune modulation and gut-brain/gut-lung interactions), and translational challenges, and identify future directions. Unlike previous reviews that mainly pool heterogeneous trials or summarize individual studies, this review applies an explicit evidence-stratification framework, distinguishing established clinical benefits, inconsistent or context-dependent outcomes, and preliminary/preclinical mechanisms. It also emphasizes strain-, dose-, gestational-age (GA)-, and product-specific interpretation, and identifies translational gaps that must be addressed before routine clinical implementation.

## 2 Gut microbiota composition and dysbiosis in preterm infants

### 2.1 Characteristics of the preterm gut microbiome

The developmental trajectory of the gut microbiome in preterm infants deviates substantially from that of healthy term infants, and this divergence begins immediately after birth (Xiang et al., 2023; Tao et al., 2026; Chen et al., 2023). In term infants born vaginally and fed with breast milk, the gut is typically colonized by a diverse array of commensal bacteria, with *Bifidobacterium* species predominating and playing a critical role in immune maturation and metabolic programming (Chen et al., 2024; Luoto et al., 2023). By contrast, preterm infants, particularly those requiring neonatal intensive care, exhibit a gut microbial ecosystem that is characterized by low alpha diversity, temporal instability, and a relative abundance of potentially pathogenic taxa (Tao et al., 2026; Chen et al., 2023; Song et al., 2026).

Multiple observational studies have systematically characterized the compositional features of the preterm gut microbiota. At the phylum level, the gut of preterm infants is often dominated by *Proteobacteria* and *Firmicutes*, with a relative paucity of *Actinobacteria*, particularly *Bifidobacterium*, and *Bacteroidetes* (Jia et al., 2022; Chen et al., 2023; Wu et al., 2025). At the genus level, *Klebsiella*, *Escherichia-Shigella*, *Enterococcus*, and *Staphylococcus* frequently constitute the core microbiota, whereas beneficial genera such as *Bifidobacterium* and *Lactobacillus* are present at substantially lower abundances compared to term infants (Chen et al., 2023; Chen et al., 2024; Chang et al., 2024). A study by Chen et al. (2023) examining 92 preterm infants in a neonatal intensive care unit (NICU) over the first 60 days of life identified the top five phyla as *Proteobacteria*, *Firmicutes*, *Actinobacteria*, *Tenericutes*, and *Bacteroidetes*, with *Proteobacteria* and *Firmicutes* accounting for the vast majority of the microbial community (Chen et al., 2023). At the genus level, *Pseudomonas*, *Staphylococcus*, and *Klebsiella* were among the most abundant taxa (Chen et al., 2023). This compositional pattern appears to be modulated by gestational age, with extremely preterm infants displaying even more pronounced deviations and lower *Bifidobacterium breve* abundance compared to moderate preterm infants (Chen et al., 2024; Devarajalu et al., 2024).

Jia et al. (2022) profiled the dynamic changes of gut microbiota in preterm infants of different gestational ages from birth to 1 year after birth, demonstrating that alpha diversity of meconium in extremely preterm infants was higher than that in very preterm, moderate to late preterm, and term groups, and that the duration of opportunistic pathogen dominance varied across gestational age groups (Jia et al., 2022). The study also found that the abundance of *Bifidobacterium* was enriched at different time points in different gestational age groups, suggesting that the timing of probiotic intervention may need to be tailored to gestational age (Jia et al., 2022). Devarajalu et al. (2024) similarly reported that gestational age and probiotic supplementation altered the gut microbiota of preterm infants admitted to the NICU, with decreased abundance of *Bifidobacterium breve* observed in extremely preterm infants compared to moderate preterm infants (Devarajalu et al., 2024).

Wu et al. (2025) in a prospective study of very low birth weight (VLBW) infants, identified 45 bacterial phyla and 1,327 genera, with *Proteobacteria* (65.64%) and *Firmicutes* (29.64%) as the dominant phyla. At the genus level, the top 10 dominant genera represented 91.9% of the total abundance, with *Klebsiella*, *Streptococcus*, and *Clostridium_sensu_stricto_1* being significantly more abundant in VLBW infants compared to controls (Wu et al., 2025). Chen et al. (2023) in a study examining gut microbiota changes in 92 NICU preterm infants in China, reported that the top five phyla accounted for 99.69% of the abundance, with *Proteobacteria*, *Firmicutes*, and *Actinobacteria* being the most abundant. The abundance of *Proteobacteria* and *Pseudomonas* showed a decreasing trend at first, reached a minimum at day 14, and then increased, while the opposite trend was observed for *Firmicutes* (Chen et al., 2023). These findings collectively indicate that the preterm gut microbiome is characterized by a distinct and dynamic compositional pattern that differs markedly from that of term infants (Figures 1, 2).

**Figure 1:**
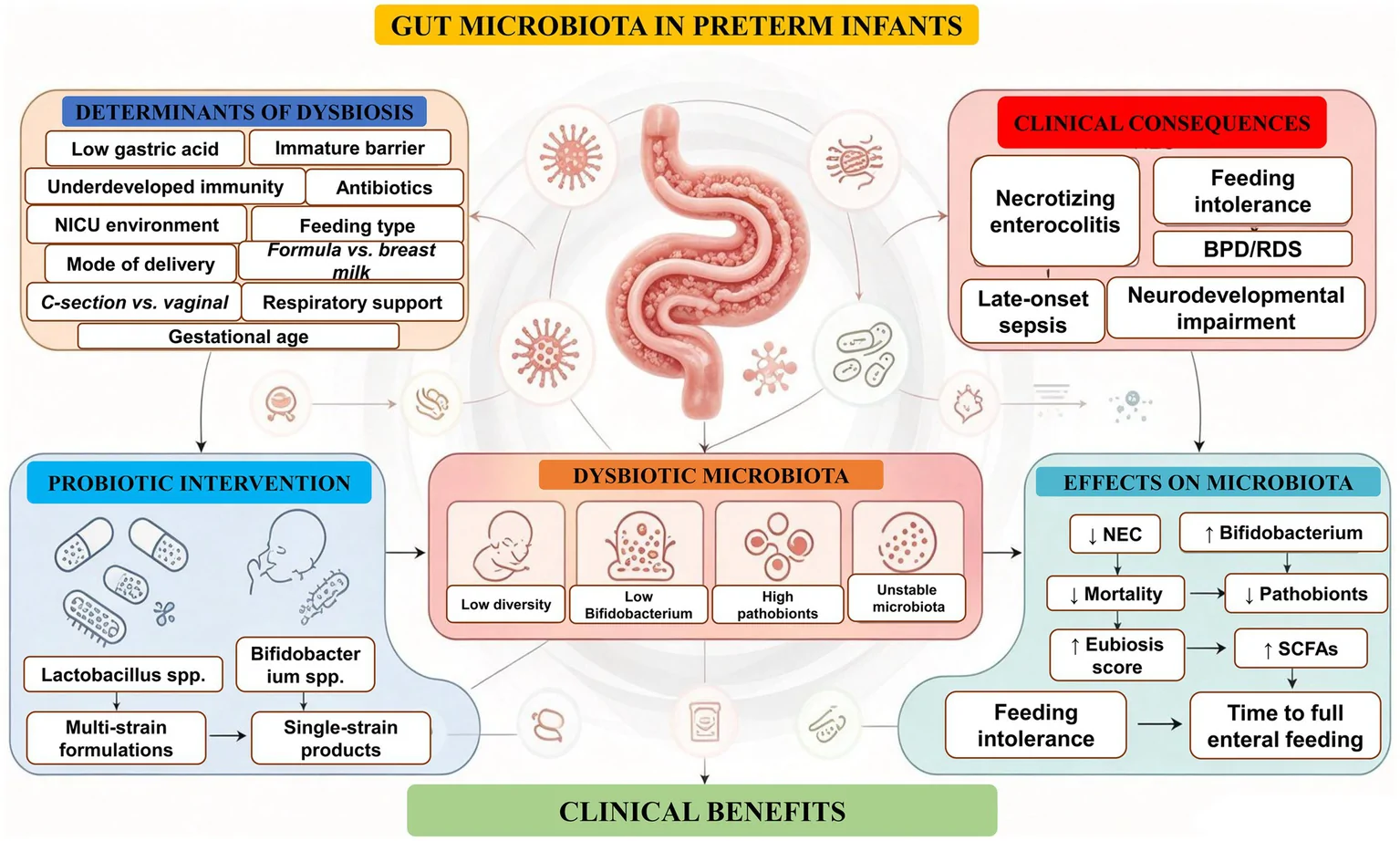
Mechanistic framework of dysbiosis pathogenesis and probiotic therapeutic effects in preterm neonates. Schematic graphical abstract depicting causal drivers of preterm intestinal dysbiosis, core microbial aberrations, associated adverse clinical outcomes, and multi-step probiotic-mediated microbiota restoration and clinical improvements. Key determinants of dysbiosis include intestinal physiological immaturity, NICU clinical interventions, feeding and delivery modes. Central dysbiotic signatures feature low microbial diversity, depleted *Bifidobacterium*, pathobiont bloom, and unstable microbiota, alongside cryptic AMR gene risks. Probiotic formulations of *Lactobacillus* and *Bifidobacterium* remodel gut microbial composition to elevate SCFA levels and re-establish eubiosis, subsequently mitigating major neonatal morbidities including NEC, feeding intolerance and BPD/RDS. Clinical benefits are stratified by evidence strength: NEC reduction represents the most robust endpoint, while neurodevelopmental gains show moderate trial evidence. All probiotic effects shown are strain- and formulation-specific and should not be generalized across products. NICU, Neonatal Intensive Care Unit; AMR, antimicrobial resistance; BPD, bronchopulmonary dysplasia; RDS, Respiratory Distress Syndrome; NEC, necrotizing enterocolitis; SCFAs, short-chain fatty acids.

**Figure 2:**
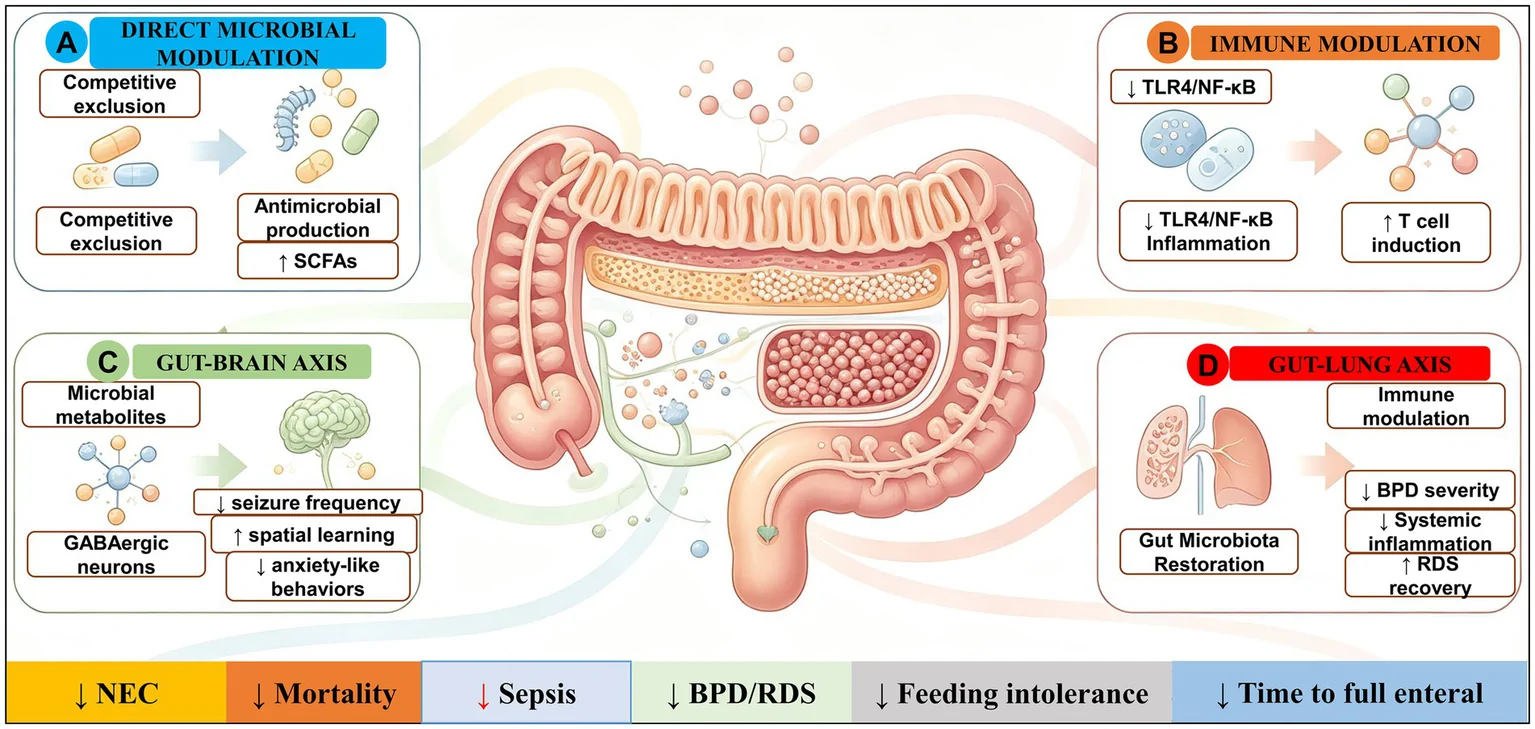
Proposed mechanisms of probiotic action and their effects on preterm infant health. Schematic illustration of the multiple mechanisms through which probiotics exert their effects in preterm infants. The figure depicts the interconnected pathways including: **(A)** direct microbial modulation (competitive exclusion, antimicrobial production, and metabolic effects); **(B)** Immune modulation (effects on innate and adaptive immunity, reduction of inflammation); **(C)** Gut-brain axis interactions (effects on neurodevelopment, behavior, and seizure prevention); and **(D)** Gut-lung axis interactions (effects on respiratory outcomes). Mechanistic pathways are strain-specific; findings from individual strains should not be extrapolated to all probiotics. SCFA production should not be attributed solely to supplemented *Bifidobacterium* and *Lactobacillus*; these strains mainly produce acetate and lactate, whereas propionate and butyrate arise from other commensal taxa or cross-feeding. Arrow color indicates the strength of evidence: Black solid arrows denote established or moderate evidence from human RCTs or meta-analyses; red solid arrows denote preliminary or preclinical evidence from animal models, *in vitro* studies, or mechanistic hypotheses. SCFAs, short-chain fatty acids; TLR4, Toll-like receptor 4; NF-κB, nuclear factor kappa B.

The developmental trajectory of the gut microbiome differs substantially across GA strata. GA is the main factor contributing to differences in meconium microbial composition (Chen et al., 2023). Extremely preterm (EP; <28 weeks) infants exhibit more pronounced deviations, including delayed colonization by obligate anaerobes and persistent dominance by facultative anaerobes, whereas very preterm (VP; 28–31 weeks) infants develop toward a *Bifidobacterium*-dominated community (Zwittink et al., 2017). Metaproteomic analyses have identified a GA-dependent microbial signature enabling differentiation between EP (25–27 weeks) and VP (30 weeks) infants. *Bifidobacterium breve* abundance is significantly lower in EP infants compared with moderate preterm infants (29–32 weeks) (Devarajalu et al., 2024). These findings suggest that the timing and nature of probiotic intervention may need to be tailored to GA (Table 1).

**Table 1:** Characteristics of gut microbiota in preterm infants compared to term infants.

Feature	Preterm infants	Term infants	Key references
Alpha diversity	Low	High	Tao et al. (2026), Jia et al. (2022), Chen et al. (2023), and Song et al. (2026)
Temporal stability	Low (highly dynamic)	High (relatively stable)	Tao et al. (2026), Jia et al. (2022), and Samara et al. (2022)
Dominant phyla	*Proteobacteria, Firmicutes*	*Actinobacteria*, *Firmicutes*, *Bacteroidetes*	Jia et al. (2022), Chen et al. (2023), and Wu et al. (2025)
*Bifidobacterium* abundance	Low (often <1%)	High (predominant genus)	Chen et al. (2023), Chen et al. (2024), Chang et al. (2024), and Butcher et al. (2018)
*Lactobacillus* abundance	Low	Moderate to high	Chen et al. (2023), Chen et al. (2024), and Chang et al. (2024)
Pathobiont abundance	High (*Klebsiella*, *Escherichia-Shigella*, *Enterococcus*, *Staphylococcus*)	Low	Jia et al. (2022), Chen et al. (2023), Song et al. (2026), and Graspeuntner et al. (2019)
*Clostridium* species	Often enriched	Low	Chen et al. (2023), Rozé et al. (2017), Song et al. (2026), and Thatrimontrichai et al. (2025)
Gestational age effect	More pronounced dysbiosis with lower GA	Not applicable	Jia et al. (2022), Chen et al. (2024), and Devarajalu et al. (2024)
Microbial maturation trajectory	Delayed, may persist for months	Rapid maturation	Tao et al. (2026), Jia et al. (2022), Luoto et al. (2023), and Samara et al. (2022)

GA, gestational age.

### 2.2 Determinants of gut dysbiosis

The determinants of gut dysbiosis in preterm infants are multifactorial and interconnected (Tao et al., 2026; Navarro-Tapia et al., 2020). Physiological immaturity of the gastrointestinal tract, including reduced gastric acid secretion, immature intestinal barrier function, and underdeveloped mucosal immunity, renders preterm infants particularly susceptible to aberrant colonization patterns (Xiang et al., 2023). Clinical interventions inherent to NICU care further compound this vulnerability. Antibiotic administration, which is nearly universal in this population, exerts a profound and sustained impact on the gut microbiota, depleting beneficial commensals and promoting the expansion of antibiotic-resistant pathobionts (Healy et al., 2025; Gong et al., 2021; Kurath-Koller et al., 2020). Healy et al. (2025) demonstrated that antibiotic exposure in very preterm infants was independently associated with significantly lower *Bifidobacterium* abundance, with an average reduction of 250-fold compared to unexposed infants. Gong et al. (2021) reported that a triple-viable powder containing *Bifidobacterium longum*, *Lactobacillus acidophilus*, and *Enterococcus faecalis* (each at 1 × 10^7^ CFU/g) administered at 1 g twice daily (2 g/day, corresponding to 6 × 10^7^ CFU/day) from day 3 after birth for 7 days increased beneficial bacteria and partially alleviated antibiotic-induced damage to microbiota composition and function.

Delivery mode also influences initial colonization. Zeber-Lubecka et al. (2016) examined the effect of mode of delivery on the intestinal microbial community in preterm infants and found that while *Firmicutes* and *Proteobacteria* remained stable during the observation period, *Actinobacteria* and *Bacteroidetes* increased in abundance, the latter particularly more sharply in vaginally delivered infants. However, Butcher et al. (2018) reported that in exclusively mother’s milk-fed VLBW infants, microbiotas did not cluster by birth mode, gestational age, or weeks after birth, and instead clustered as a function of patient identity. This finding suggests that while delivery mode may influence early colonization patterns, other factors, particularly feeding type, may play a more dominant role in shaping the microbiota over time.

Vaginal seeding as a test of microbial restoration. A conceptually important question is whether restoring maternal microbial exposure after cesarean delivery can partially normalize infant microbiome development. This has been explored through “vaginal seeding” (also termed vaginal microbiota transfer), in which the neonate is exposed to maternal vaginal fluids at birth. A proof-of-concept study demonstrated that the stool microbiota of cesarean-delivered infants could be partially modified to resemble that of vaginally delivered infants through this procedure (Dominguez-Bello et al., 2016). A systematic review of eight studies involving 558 cesarean-born infants (274 exposed to vaginal seeding) concluded that vaginal seeding facilitates partial restoration of key microbial communities, with increased relative abundance of *Bacteroides* and *Lactobacillus*, but does not fully replicate natural microbial transfer at birth (Wang et al., 2024). Conflicting results regarding microbial diversity and limited evidence linking these microbial changes to clinical outcomes were noted.

With respect to clinical outcomes, the ACTIVATE trial, a randomized, double-blind, placebo-controlled pilot study of 114 mother-infant pairs, found no difference in food allergen sensitization at 12 months between vaginally seeded and placebo-seeded cesarean-born infants, although no adverse events related to vaginal seeding were reported when strict infectious disease screening criteria were followed (Tsuang et al., 2025). A pilot randomized controlled trial (RCT) from southern China reported that VMT was associated with improved social–emotional scores at 12 months, but the neurodevelopmental benefits observed at 6 months appeared to diminish over time (Zhou et al., 2026).

Despite these preliminary signals, vaginal seeding is not currently recommended by major healthcare organizations, including the American College of Obstetricians and Gynecologists and the Danish Society of Obstetrics and Gynecology, due to safety concerns and insufficient evidence (American College of Obstetricians and Gynecologists (ACOG), 2017; Haahr et al., 2018). The Danish Society is strongly against the practice in preterm infants (gestational age <37 weeks) because prematurity substantially increases the risk of neonatal infection. Potential pathogens transmissible through vaginal seeding include group *B Streptococcus*, *Escherichia coli*, *herpes simplex virus*, and *Chlamydia trachomatis*, and a case of neonatal herpes simplex infection following vaginal seeding has been reported. It is important to distinguish vaginal seeding from probiotic supplementation: unlike probiotics, which use defined, characterized strains with established safety profiles, vaginal seeding involves transfer of an uncharacterized maternal microbiota with potential pathogens. These safety considerations currently preclude vaginal seeding in preterm infants outside of approved research protocols.

Feeding type is a critical determinant of gut microbiota composition in all neonates and is particularly important in preterm infants, in whom immature gut function, delayed establishment of enteral feeding, and frequent NICU-related exposures may amplify its effects. Xu et al. (2018), in a systematic review of preterm infants, reported that mother’s own breast milk feeding influenced the compositional structure of preterm infants’ gut microbial community and increased diversity compared to donor human milk and formula feeding. Although the evidence summarized below focuses on preterm infants, the principle that feeding type shapes early microbial colonization applies to neonates in general. Yang et al. (2024) found that preterm infants with >70% in-hospital breast milk intake had lower alpha diversity compared to those with ≤70% intake, which may be related to delayed maturation of gut microbes and represents a more developmental gut time window. Hattoufi et al. (2026) in a study of Moroccan preterm newborns, reported that *Lactobacillus* spp. was only detected in infants fed by both breast milk and formula milk over the first 2 weeks of life, and that *Clostridium difficile* and *Clostridium perfringens* were found in 83% of formula-fed newborns in the second week of life and in all newborns by the end of the third week. Chen et al. (2023) reported that the abundance of *Proteobacteria* at the phylum level and *Streptococcus* at the genus level in formula-fed infants were significantly higher than in breast-fed infants.

Other factors contributing to dysbiosis include the NICU environment itself, which may expose infants to hospital-acquired pathogens, and the use of non-invasive positive pressure ventilation, which has been associated with suppression of diversity accrual and obligate anaerobic growth in the preterm intestine (Hutchinson et al., 2024). Hutchinson et al. (2024) reported that bacterial diversity increased at the point of transitioning off non-invasive positive pressure respiratory support, accompanied by a significant increase in anaerobic genera (*Bifidobacteria* spp.*, Veillonella* spp.) and a non-significant fall in *Enterobacteriaceae*.

Beyond antibiotics, delivery mode, feeding type, and respiratory support, several other NICU-related exposures warrant consideration as determinants of gut dysbiosis in preterm infants. Among these, postnatal opioid exposure is common in hospitalized preterm infants for analgesia and sedation, and accumulating evidence suggests it may adversely affect the developing gut microbiome. A recent study by Thänert et al. (2024) demonstrated that fentanyl exposure during NICU hospitalization is associated with decreased fecal microbiome richness, negative association with the microbial taxa *Actinobacteriota* and *Bacteroidia*, increased constipation, elevated inflammation-related metabolites, and altered microbial virulence. Increased duration of postnatal fentanyl exposure was associated with worse cognitive scores on the Bayley Scales at 2 years and lower intelligence quotient at 3–8 years of age, with virulence factors present in the microbiome mediating the association between fentanyl and cognitive outcomes at 2 years (Thänert et al., 2024). Preclinical studies in newborn mice further reveal that early opioid exposure (morphine) significantly alters gut microbial composition, leading to a substantial reduction in beneficial commensal species (Antoine et al., 2022). These findings highlight the gut-brain axis connection in NICU patients and suggest that opioid exposure represents an underrecognized determinant of dysbiosis (Table 2).

**Table 2:** Additional NICU-related exposures influencing gut microbiota development in preterm infants.

Exposure	Effect on gut microbiota	Clinical/outcome implications	Key references
Fentanyl (opioid)	↓ Fecal microbiome richness; ↓ Actinobacteriota, Bacteroidia; ↑ constipation; ↑ inflammation-related metabolites; altered virulence	Worse cognitive scores at 2 years; lower IQ at 3–8 years	Thänert et al. (2024)
Morphine (opioid, preclinical)	Altered gut microbial composition; ↓ beneficial commensals	Potential long-term neurobehavioral effects	Antoine et al. (2022)
Maternal diet (high fruits/vegetables)	↑ Microbial diversity; ↑ *Cutibacterium*, *Parabacteroides*, *Lactococcus*; ↓ pro-inflammatory bacteria	Beneficial microbial composition in infant gut	Catassi et al. (2024)
Maternal high-fat diet/obesity	Shorter breastfeeding duration; altered infant microbiome	Potential adverse impact on infant health	Le Bourgot et al. (2024)
PPIs	↓ *Lactobacillus*, *Stenotrophomonas*; ↑ *Haemophilus*; altered Firmicutes/Bacteroidetes/Proteobacteria	↑ NEC, late-onset sepsis, *C. difficile* infection	De Rose et al. (2024) and Jeong (2022)
H2 blockers	↑ Proteobacteria over Firmicutes	↑ NEC risk	La Rosa et al. (2014)
Prolonged hospitalization	Delayed microbiota maturation; ↓ stability	Hindered establishment of term-like microbiota	Qian et al. (2024) and Li et al. (2025)
Enteral feeding tubes	Microbial biofilm formation	Potential source of microbial colonization	Brooks et al. (2018)

PPI, proton pump inhibitor; NEC, necrotizing enterocolitis; IQ, intelligence quotient.

Maternal factors also play an important role. Maternal diet during pregnancy and lactation significantly influences infant gut microbiota development. Higher maternal consumption of fruits and vegetables is associated with a more diverse and beneficial microbial composition in the infant gut, especially in those who are delivered vaginally and exclusively breastfed, and is linked to increased counts of beneficial bacteria such as *Cutibacterium*, *Parabacteroides*, and *Lactococcus*, along with reduced counts of potentially pro-inflammatory bacteria (Catassi et al., 2024). Mothers with a high intake of fibers and plant proteins tend to have breast milk enriched with beneficial bacteria like *Bifidobacterium* (Catassi et al., 2024). Maternal prebiotic intake may facilitate the transfer of gut-derived short-chain fatty acids (SCFAs) into milk, potentially supporting infant gut health (Le Bourgot et al., 2024). Conversely, maternal high-fat diet and obesity are associated with shorter breastfeeding durations and potentially impact the infant’s microbiome development (Catassi et al., 2024) (Table 2).

In addition to opioids and maternal diet, acid-suppressive medications are frequently prescribed in NICUs for gastroesophageal reflux and feeding intolerance, and they may also disturb the developing microbiome. Proton pump inhibitor (PPI) use creates dysbiosis of the microbiome in the mouth, gut, and lungs (De Rose et al., 2024). PPI usage for 8 weeks resulted in a decrease in *Lactobacillus* and *Stenotrophomonas* and an increase in *Haemophilus*, with significant changes in the relative abundances of *Firmicutes*, *Bacteroidetes*, and *Proteobacteria* (Jeong, 2022). Specific adverse effects associated with PPIs include NEC, LOS in premature infants, and *Clostridium difficile* infection (Jeong, 2022). H2 blockers favor the proliferation of *Proteobacteria* over *Firmicutes*, and retrospective database reviews and secondary analysis of large multi-center studies show that the use of antacids (both H2 blockers and PPIs) increases the risk for NEC development (La Rosa et al., 2014). Given that both drug categories and the NICU stay can lead to intestinal dysbiosis, their use should be minimized in neonates (Table 2).

Finally, beyond specific medications and maternal factors, the NICU environment itself and the length of hospitalization substantially shape gut microbiome dynamics. A recent large-scale study analyzing over 2,500 fecal samples with metagenomics and metatranscriptomics demonstrated that medical interventions in the NICU shape gut microbiome dynamics in the first months of life (Qian et al., 2024). Prolonged hospitalization affects the development and stability of the gut microbiota in preterm infants, hindering their ability to establish a gut microbiota resembling that of term infants (Li et al., 2025). Enteral feeding tubes commonly used in the NICU rapidly develop microbial biofilms that may contribute to microbial colonization (Brooks et al., 2018) (Table 2).

The impact of these exposures is GA-dependent: the younger the GA, the more likely preterm infants are to receive antibiotics and invasive procedures, compounding the risk of dysbiosis (Li et al., 2025). GA and its associated intensity of care (e.g., antibiotics and respiratory support) affect intestinal microbiota composition and activity, potentially setting the stage for early and later life health complications (Zwittink et al., 2017).

### 2.3 Clinical consequences of dysbiosis

The clinical consequences of dysbiosis in preterm infants extend beyond the gastrointestinal tract. A substantial body of evidence has linked gut microbial composition to the pathogenesis of NEC, one of the most devastating conditions affecting preterm infants (Pei et al., 2025; Rozé et al., 2017; Feng et al., 2026). Rozé et al. (2017) in a large nationwide study, found that colonization with *Clostridium neonatale* and *Staphylococcus aureus* was significantly associated with NEC development. Similarly, Song et al. (2026) in a retrospective cohort study, reported that preterm infants who developed NEC had significantly reduced gut microbiota diversity, lower relative abundances of *Bifidobacterium* and *Lactobacillus*, and increased abundances of *Enterobacteriaceae* and *Klebsiella* at 14 days after birth compared to controls. Li and Peng further demonstrated that gut microbiota characteristics were associated with surgical intervention status in NEC, with lower abundances of *Bifidobacterium* and higher abundances of *Proteobacteria* and *Clostridium* independently associated with the need for surgery (Li and Peng, 2026). The combined model incorporating *Firmicutes*, *Proteobacteria*, *γ-Proteobacteria*, and *Clostridium* achieved an area under the curve of 0.904 for distinguishing surgical NEC from non-surgical NEC (Li and Peng, 2026).

Beyond NEC, gut dysbiosis has been implicated in other morbidities. Graspeuntner et al. (2019) reported that preterm infants who developed LOS exhibited a decelerated development of microbial diversity and specific gut microbiome signatures preceding disease onset, including higher abundance of *Bacilli* and a lack of *anaerobic bacteria*. Infants with sepsis also demonstrated a significantly different longitudinal trajectory of microbiome development, with lower *Bifidobacterium* and higher *Escherichia-Shigella* abundance observed prior to sepsis onset (Healy et al., 2025). Microbial load, as estimated by total copy number per gram, was significantly different between infants with and without sepsis, with the latter having more rapid increase and overall higher microbial load (Healy et al., 2025).

Feeding intolerance, a common complication in preterm infants, has been associated with reduced microbial diversity, decreased abundance of beneficial bacteria, and increased abundance of pathogenic bacteria (Hu et al., 2021). Hu et al. (2021) reported that the abundance levels of phylum *Proteobacteria*, class *Gammaproteobacteria*, and genera such as *Escherichia/Shigella* were higher in the feeding intolerance group than in the feeding tolerance group, while *Firmicutes*, *Negativicutes*, and *Veillonella* were higher in the feeding tolerance group. Fu C. et al. (2025) further demonstrated that *Citrobacter* and *Acinetobacter* were, respectively, involved in feeding intolerance in preterm infants of different gestational ages, with *Citrobacter* implicated in infants with gestational age <32 weeks and *Acinetobacter* implicated in those with gestational age ≥32 weeks.

Bronchopulmonary dysplasia has also been linked to gut microbial alterations and inflammatory responses. Zhang et al. (2022) found that the BPD group had high levels of Interleukin (IL)-1β, IL-4, IL-6, IL-8, and tumor necrosis factor alpha (TNF-α), whereas IL-10 was decreased, and the Shannon diversity index of the BPD group was lower. The relative abundances of *Proteobacteria* in the BPD group increased significantly from days 3–7 to days 14–28, while *Firmicutes* decreased (Zhang et al., 2022). Fu Y. et al. (2025) reported that the gut microbiota diversity in the neonatal respiratory distress syndrome (RDS) group was significantly greater than in the neonatal non-RDS group, and that probiotic intervention notably restored the gut microbiota structure, particularly by enhancing the abundance of beneficial genera such as *Streptococcus*, *Bifidobacterium*, and *Clostridium*. Seo et al. (2026) characterized RDS-associated gut dysbiosis and its partial resolution following surfactant replacement therapy, and established proof-of-concept for a disease-specific probiotic approach.

## 3 Effects of probiotic supplementation on gut microbiota composition and function

The magnitude and persistence of probiotic effects depend not only on strain identity but also on daily dose, duration of administration, viability at administration, and delivery vehicle. However, these parameters are inconsistently reported across trials, limiting direct comparison. In this section, we report daily dose and duration where available; studies that did not report these parameters are explicitly identified.

### 3.1 Modulation of bacterial composition

Given the central role of dysbiosis in preterm infant morbidity, probiotic supplementation has been extensively investigated as a strategy to restore a more favorable microbial ecosystem. A consistent finding across numerous studies is that probiotic administration increases the intestinal abundance of the supplemented bacterial genera, particularly *Bifidobacterium* and *Lactobacillus* (Chang et al., 2024; Samara et al., 2022; Plummer et al., 2018; Alcon-Giner et al., 2020; Mohan et al., 2006; Moreno-Sanz et al., 2022; Abdulkadir et al., 2016; Qiao et al., 2017). In a RCT involving 71 premature infants, Wang H. et al. (2025) found that supplementation with *Bifidobacterium animalis* subsp. *lactis BB-12* (DiPro; Chr. Hansen A/S, Denmark) at a daily dose of 1 × 10^9^ CFU/day (6 drops/day, each 6-drop dose containing 1 billion CFU), mixed with breast milk or formula and interrupted during fasting, was associated with increased levels of amino acids in the gut and reduced serum inflammatory markers. The intervention was evaluated at postnatal weeks 2 and 4; the total duration of supplementation was not explicitly reported. Chang et al. (2024) in a study of VLBW preterm infants, reported significantly higher relative abundances of *Bifidobacterium* and *Lactobacillus* in the probiotic group on days 14, 30, and 60 after birth compared to controls. The probiotic group also exhibited a significantly lower proportion of participants with low abundance (<1%) of *Bifidobacterium* or *Lactobacillus* on days 14, 30, and 60 compared to the control group (Chang et al., 2024).

Samara et al. (2022) in extremely preterm infants, demonstrated that a four-strain *Bifidobacterium* product accelerated the transition to a mature, term-like microbiome with higher stability and species interconnectivity. The study reported that *Bifidobacterium* strains and stool metabolites were the best predictors of microbiome maturation, and structural equation modeling confirmed probiotics as a major determinant for the trajectory of microbiome assembly (Samara et al., 2022). *Bifidobacterium*-driven microbiome maturation was also linked to an anti-inflammatory intestinal immune milieu (Samara et al., 2022).

The ProPrems trial sub-study by Plummer et al. (2018) found that probiotic supplementation with *B. longum subsp. infantis BB-02, B. animalis subsp. lactis BB-12*, and *S. thermophilus TH-4* (ProPrems, one sachet per day (>2 × 10^14^ bacteria per sachet), until 34 weeks corrected gestational age), was associated with higher abundance of *Bifidobacterium* (adjusted odds ratio [OR] 17.22) and reduced abundance of *Enterococcus*. Abdulkadir et al. (2016) using Infloran® (*Lactobacillus acidophilus and Bifidobacterium bifidum; strain designations according to product specification*), reported greater *Bifidobacterium* (15.1%) and *Lactobacillus* (4.2%) during supplementation compared to controls (4.0 and 0%, respectively). While *Lactobacillus* became reduced after the probiotic had been stopped, *Bifidobacterium* remained high following discharge, suggestive of successful colonization (Abdulkadir et al., 2016).

Moreno-Sanz et al. (2022) evaluated a new probiotic strain combination (*Ligilactobacillus salivarius subsp. infantis PS11603* [1 × 10^9^ CFU/day] and *Bifidobacterium longum PS10402[1 × 10^8^ CFU/day]*) and found that a higher number of participants were colonized by *Lactobacilli* in the probiotic group than in the placebo group (93% vs. 46%), with similar results for *bifidobacterial* colonization (100% vs. 69%). Earlier colonization was observed in the probiotics group versus the placebo group, specifically 5 weeks for *Lactobacillus* and 1 week for *Bifidobacterium* (Moreno-Sanz et al., 2022). Chang et al. (2022), in a prospective cohort of 120 extremely preterm infants (GA ≤ 28 weeks), reported that routine daily supplementation with Infloran (*Lactobacillus acidophilus* and *Bifidobacterium bifidum*; one 250 mg capsule once daily, started soon after the initiation of oral feeding at a median of 8 days of life; CFU/day not reported) was associated with an increased abundance of *Lactobacillus* (adjusted OR 4.33; 95% CI 1.89–9.96; *p* = 0.009) compared with the control group.

However, the effects of probiotics on the gut microbiota are not uniformly positive or consistent across all studies. van Best et al. (2020), in a longitudinal observational study of 80 preterm infants (GA < 32 weeks), found that daily supplementation with either Infloran (250 mg/day; *Lactobacillus acidophilus ATCC 4356* + *Bifidobacterium longum subsp. infantis ATCC 15697*, each at 1 × 10^9^ CFU/day; total 2 × 10^9^ CFU/day) or DarmfloraPlus (250 mg/day; *L. acidophilus La-14, B. longum subsp. longum Bl-05*, *L. casei* Lc-11, and *B. animalis subsp. lactis*, each at 1 × 10^9^ CFU/day; total 4 × 10^9^ CFU/day), continued until 36 weeks postmenstrual age, was associated with higher relative abundance of *Bifidobacterium* and *Enterobacter* and reduced abundance of *Escherichia*, *Enterococcus*, and *Klebsiella*. Colonization by probiotic bifidobacteria was observed in only approximately 50% of infants and remained transient in the majority of cases. The PRIMAL trial, a large multicenter RCTs, demonstrated that a multi-strain product containing *Bifidobacterium animalis subsp. lactis BB-12*, *Bifidobacterium longum subsp. infantis*, and *Lactobacillus acidophilus La-5*, administered at 1.5 × 10^9^ CFU of each strain per day (once daily for 28 days, started within the first 72 h of life), increased eubiosis scores—a composite measure of microbiome similarity to healthy term infants—at both the genus and species levels, but did not reduce colonization with MDRO+ (Van Rossum et al., 2024). At the genus level, the probiotic group had higher eubiosis scores (median 0.47 vs. 0.41; OR 1.07), and at the species level, the difference was more pronounced (median 0.87 vs. 0.59; OR 1.28) (Van Rossum et al., 2024). Environmental uptake of the *B. infantis* probiotic strain in the control group was common (41 of 84, 49%), which was highly variable across sites and particularly occurred in infants with a sibling who was treated with probiotics (Van Rossum et al., 2024).

Birth GA has a significant impact on probiotic colonization. In a prospective analysis of 52 preterm infants, bifidobacterial colonization always occurred in infants born at a GA greater than 32.9 weeks (*p* < 0.05), and birth GA acted as a major determinant of bifidobacterial colonization (Butel et al., 2007). In a study by Devarajalu et al. (2024) probiotic supplementation increased *Bifidobacterium* regardless of GA, but EP infants showed reduced *B. breve* abundance compared with moderate preterm infants. Probiotic administration rendered the gut microbiota and resistome of EP infants more similar to term infants on day 7 and ameliorated GA-driven loss of microbiota interconnectivity and stability. These findings indicate that GA should be considered when selecting probiotic strains and timing (Tables 3, 4).

**Table 3:** Summary of probiotic effects on gut microbiota composition in preterm infants.

Microbiota parameter	Effect of probiotic supplementation	Consistency of evidence	Key references
*Bifidobacterium* abundance	Increased	High	Chang et al. (2024), Samara et al. (2022), Plummer et al. (2018), Alcon-Giner et al. (2020), Mohan et al. (2006), Moreno-Sanz et al. (2022), Abdulkadir et al. (2016), Qiao et al. (2017), and van Best et al. (2020)
*Lactobacillus* abundance	Increased	High	Chang et al. (2024), Moreno-Sanz et al. (2022), Abdulkadir et al. (2016), Qiao et al. (2017), and Chang et al. (2022)
*Enterococcus* abundance	Decreased	Moderate	Plummer et al. (2018), van Best et al. (2020), and Ye et al. (2026)
*Klebsiella* abundance	Decreased	Moderate	Chang et al. (2024), Mohan et al. (2006), van Best et al. (2020), and Ye et al. (2026)
*Escherichia-Shigella* abundance	Decreased	Moderate	Chang et al. (2024) and Healy et al. (2025)
*Clostridium* spp. abundance	Decreased	Moderate	Mohan et al. (2006)
Microbial diversity	Increased or unchanged	Moderate	Kurath-Koller et al. (2020), Samara et al. (2022), Moreno-Sanz et al. (2022), and Athalye-Jape et al. (2025)
Microbial stability	Increased	Moderate	Samara et al. (2022), van Best et al. (2020), and Ye et al. (2026)
Fecal acetate/lactate	Increased	Moderate	Alcon-Giner et al. (2020)
Fecal pH	Decreased	Moderate	Alcon-Giner et al. (2020)
Fecal calprotectin	Decreased (strain-dependent)	Moderate	Wang H. et al. (2025), Larke et al. (2022), and Athalye-Jape et al. (2025)
MDRO + colonization	No significant reduction	High	Van Rossum et al. (2024)
Eubiosis score	Increased	High	Van Rossum et al. (2024)

MDRO+, multidrug-resistant organisms or highly epidemic bacteria.

**Table 4:** Strain-specific probiotic interventions, daily dose, duration, and outcomes in preterm infants.

Study	Probiotic strain(s) with full designation	Daily dose (CFU/day)	Duration	Population (GA/BW)	Key outcomes	Dose reported?
Wang H. et al. (2025)	*Bifidobacterium animalis* subsp. *lactis* BB-12 (DiPro; Chr. Hansen A/S, Denmark)	1 × 109 CFU/day (6 drops/day; each 6-drop dose contains 1 billion CFU)	Not explicitly stated; evaluated at postnatal weeks 2 and 4	GA ≤ 32 weeks, BW < 1,500 g (*n* = 71)	↑ Gut amino acids; ↓ TLR4/NF-κB, IL-1β, TNF-α; ↓ feeding intolerance	Yes
Chang et al. (2022)	*Lactobacillus acidophilus* + *Bifidobacterium bifidum* (Infloran; strain designations not reported)	One 250 mg capsule once daily; CFU/day not reported	Started soon after oral feeding (median day 8; IQR 6–12); ongoing at stool collection (median day 14); total duration not stated	Extremely preterm infants, GA ≤ 28 and >24 weeks; *n* = 120 (70 probiotic, 50 control)	↑ *Lactobacillus* (AOR 4.33); ↓ LOS (47.1% vs. 70.0%); ↓ TPN days; no significant NEC difference	Partial
Gong et al. (2021)	*Bifidobacterium longum* + *Lactobacillus acidophilus* + *Enterococcus faecalis* (Bifid triple viable powder)	Each strain 1 × 107 CFU/g; 1 g twice daily = 2 g/day; total 6 × 107 CFU/day	7 days, started on day 3 after birth	GA 28–37 weeks, cesarean section, formula-fed (*n* = 40)	↑ Beneficial bacteria; ↑ cellular community prokaryotes; alleviated antibiotic-induced microbiota damage	Yes
Plummer et al. (2018)	*B. longum* subsp. *infantis* BB-02 + *B. animalis* subsp. *lactis* BB-12 + *Streptococcus thermophilus* TH-4 (ProPrems)	>2 × 1014 CFU per sachet; 1 sachet/day	Until 34 weeks corrected gestational age or 4–6 weeks	Preterm infants (ProPrems sub-study)	↑ *Bifidobacterium* (aOR 17.22); ↓ *Enterococcus*	Yes
Moreno-Sanz et al. (2022)	*Ligilactobacillus salivarius* subsp. *infantis* PS11603 + *Bifidobacterium longum* PS10402	1 × 109 CFU/day (PS11603) + 1 × 108 CFU/day (PS10402)	Once daily until 36 weeks postmenstrual age or discharge	GA 28⁺^0^–30⁺^6^ weeks (*n* = 30)	↑ Colonization by both strains; earlier colonization vs. placebo	Yes
Larke et al. (2022)	*Bifidobacterium longum* subsp. *infantis* EVC001 vs. *Lactobacillus reuteri* DSM 17938	8 × 109 CFU/day (EVC001) vs. 1 × 109 CFU/day (DSM 17938)	Until discharge (minimum 7 days)	Human milk-fed preterm infants	↑ Bifidobacterial fermentation; ↓ fecal calprotectin (EVC001 vs. DSM 17938)	Yes
Mohan et al. (2006)	*Bifidobacterium animalis* subsp. *lactis* Bb12	2 × 109 CFU/day	21 days	Preterm infants	↓ Enterobacteriaceae; ↓ *Clostridium* spp.	Yes
Athalye-Jape et al. (2025)	*Bifidobacterium breve* M-16 V + *B. longum* subsp. *infantis* M-63 + *B. longum* subsp. *longum* BB536 (live or heat-inactivated)	3 × 109 CFU/day (total)	3 weeks	Very preterm infants, GA < 32 weeks (*n* = 86; 43 live vs. 43 heat-inactivated)	↑ Propionate (both groups); ↑ Butyrate (heat-inactivated); no probiotic sepsis; comparable fecal calprotectin	Yes
Ye et al. (2026)	*Bacillus subtilis* + *Enterococcus faecium* (live combined preparation)	1.5 × 108 CFU/day	14 days	Antibiotic-exposed preterm infants	↓ Feeding intolerance; ↓ potentially pathogenic bacteria	Yes
Van Rossum et al. (2024)	*Bifidobacterium animalis* subsp. *lactis* BB-12 + *B. longum* subsp. *infantis* + *Lactobacillus acidophilus* La-5	1.5 × 109 CFU of each strain per day (total 4.5 × 109 CFU/day); once daily in single-dose capsule	28 days or until discharge; started within first 72 h of life	GA 28⁺^0^–32⁺^6^ weeks; *n* = 618 with day-30 follow-up	↑ Eubiosis score (genus and species level); no ↓ MDRO+ colonization (37.4% vs. 37.5%); no significant LOS difference (2.5% vs. 3.7%)	Yes
van Best et al. (2020)	P1 (Infloran): *L. acidophilus* ATCC 4356 + *B. longum* subsp. *infantis* ATCC 15697; P2 (DarmfloraPlus): *L. acidophilus* La-14, *B. longum* subsp. *longum* Bl-05, *L. casei* Lc-11, *B. animalis* subsp. *lactis*	P1: total 2 × 109 CFU/day (each strain 1 × 109 CFU/day); P2: total 4 × 109 CFU/day (each strain 1 × 109 CFU/day); both 250 mg/day once daily	Until 36 weeks postmenstrual age	Preterm infants, GA < 32 weeks (*n* = 80)	↑ *Bifidobacterium* and *Enterobacter*; ↓ *Escherichia*, *Enterococcus*, *Klebsiella*; ~50% colonization by probiotic bifidobacteria; ↓ monthly NEC incidence	Yes
Kurath-Koller et al. (2020)	Center G: *Lactobacillus rhamnosus*; Center K: *Bifidobacterium infantis* + *Lactobacillus acidophilus*; Center L: none	Center G: 1 × 109 CFU/day (split into 2 doses); Center K: 2 × 109 CFU/day each strain (total 4 × 109 CFU/day)	First 2 weeks of life (study observation period); total duration not explicitly reported	VLBW infants (<1,500 g), 3 centers, *n* = 54	Earlier increase in bacterial load; center-specific clustering; individual non-response	Yes (Centers G and K)

CFU, colony-forming units; GA, gestational age; BW, birth weight; VLBW, very low birth weight; PMA, postmenstrual age; CGA, corrected gestational age; AOR, adjusted odds ratio; LOS, late-onset sepsis; NEC, necrotizing enterocolitis; TPN, total parenteral nutrition; MDRO+, multidrug-resistant organisms or highly epidemic bacteria; P1, probiotic 1; P2, probiotic 2.

### 3.2 Metabolic and functional changes

The metabolic consequences of probiotic supplementation have also been characterized. Alcon-Giner et al. (2020) in a large observational study of 234 preterm infants, reported that supplementation with *Bifidobacterium* and *Lactobacillus* resulted in higher fecal acetate and lactate levels and lower fecal pH, consistent with the metabolic activity of the supplemented *Bifidobacterium* strain in utilizing human milk oligosaccharides (HMOs). Fecal acetate positively correlated with relative abundance of *Bifidobacterium*, and the study demonstrated that microbiota supplementation was associated with a *Bifidobacterium*-dominated preterm microbiota and gastrointestinal environment more closely resembling that of full-term infants (Alcon-Giner et al., 2020).

Larke et al. (2022) compared two probiotic strains, *Bifidobacterium longum subsp. infantis EVC001* (*B. infantis EVC001*, 8 × 10^9^ CFU/day, until discharge at minimum 7 days) and *Lactobacillus reuteri DSM 17938* (*L. reuteri DSM 17938*, 1 × 10^9^ CFU/day until discharge at minimum 7 days), and found that *B. infantis EVC001* supplementation was associated with increased fecal metabolites linked to bifidobacterial fermentation and reduced intestinal inflammation, as measured by fecal calprotectin, compared to *L. reuteri. Fecal* HMOs were significantly higher in *L. reuteri*-fed babies despite similar HMOs content in the milk consumed, and fecal calprotectin was negatively associated with the microbial metabolite indole-3-lactate (Larke et al., 2022). This study demonstrated that supplementing an HMO-catabolizing *Bifidobacterium* probiotic results in increased microbial metabolism of milk oligosaccharides and reduced intestinal inflammation relative to a noncatabolizing *Lactobacillus* probiotic in human milk-fed preterm infants (Larke et al., 2022).

Wang H. et al. (2025) reported that administration of *Bifidobacterium animalis* subsp. *lactis BB-12* at 1 × 10^9^ CFU/day (6 drops/day) was associated with increasing the levels of glutamine, glutamic acid, and kynurenine in the gut of premature infants, and associated with reducing the levels of TLR4 and nuclear factor kappa B (NF-κB) in the serum, further decreasing the secretion of the pro-inflammatory factors IL-1β and TNF-α, and alleviating systemic inflammatory reactions, thereby reducing the incidence of feeding intolerance. Athalye-Jape et al. (2025) in a RCT of 86 very preterm infants (gestational age <32 weeks), compared a live versus heat-inactivated three-strain *Bifidobacterium* mixture (*B. breve M-16 V, B. longum* subsp. *infantis M-63*, and *B. longum* subsp. *longum BB536*; total 3 × 10^9^ CFU/day for 3 weeks) and found that propionate was significantly raised in both groups, while butyrate was significantly raised in the heat-inactivated probiotic group. Bacterial richness and diversity increased but was comparable between the groups, and beta diversity showed similar community structures in both groups (Athalye-Jape et al., 2025).

### 3.3 Suppression of pathogenic taxa

Probiotic supplementation has also been shown to suppress potentially pathogenic taxa. The study by Chang et al. (2024) demonstrated significantly lower abundance of *Klebsiella* on days 14 and 30 (2.4% vs. 11.6 and 7.9% vs. 16.6%) and of *Escherichia-Shigella* on day 60 (6.1% vs. 12.3%) in the probiotic group compared to controls. Mohan et al. (2006) in an early randomized trial of *Bifidobacterium animalis* subsp. *lactis Bb12* supplementation (2 × 10^9^ CFU/day for 21 days), found reduced viable counts of *Enterobacteriaceae* (log10 values: probiotic 7.80 vs. placebo 9.03) and *Clostridium* spp. (probiotic 4.89 vs. placebo 5.99) in the probiotic group.

Kurath-Koller et al. (2020) observed that probiotics initiated an earlier increase in bacterial load, although the response was marked by individual divergences and center-specific clustering. In that multicenter cohort, Center G administered *Lactobacillus rhamnosus* at 1 × 10^9^ CFU/day orally, split into two doses per day, whereas Center K administered *Bifidobacterium infantis* (2 × 10^9^ CFU/day) plus *Lactobacillus acidophilus* (2 × 10^9^ CFU/day) in combination orally (total 4 × 10^9^ CFU/day); Center L used no probiotics. The study reported that without probiotics administration, *Lactobacillus* and *Bifidobacterium* contributed only marginally to the fecal microbiome, and some infants did not respond to probiotic administration (Kurath-Koller et al., 2020). Ye et al. (2026) reported that supplementation with a live combined preparation of *Bacillus subtilis* and *Enterococcus faecium* (1.5 × 10^8^ CFU/day for 14 days) was associated with suppression of potentially pathogenic bacteria, including *Enterococcus* and *Klebsiella*, alongside preservation of beneficial genera such as *Bifidobacterium* and *Lactobacillus*. These microbial shifts were accompanied by a more stable and mature gut microbiota profile, and the intervention was well-tolerated with no treatment-related adverse events reported (Ye et al., 2026).

However, the effects of probiotics on pathogenic bacteria are not consistently observed. Chrzanowska-Liszewska et al. (2012) found that while *L. rhamnosus GG* supplementation led to rapid growth of *L. rhamnosus GG* in the gut of bottle-fed infants, it did not decrease the amount of pathogenic organisms, nor increase weight gain during enteral feeding, or decrease length of hospital stay. The PRIMAL trial found that a multi-strain probiotic formulation (*B. animalis* subsp. *lactis BB-12, B. longum* subsp. *infantis*, and *L. acidophilus La-5*; 1.5 × 10^9^ CFU of each strain per day for 28 days) did not reduce the incidence of MDRO+ colonization at day 30 of life in preterm infants, despite modulating their microbiome toward eubiosis (Van Rossum et al., 2024).

## 4 Clinical outcomes associated with probiotic supplementation

### 4.1 NEC

The prevention of NEC is arguably the most extensively studied clinical outcome in the context of probiotic supplementation for preterm infants. Multiple systematic reviews and meta-analyses have consistently reported a significant reduction in the incidence of severe NEC (Bell stage ≥2) with probiotic use (Wang et al., 2023; Aceti et al., 2015; Morgan et al., 2020; Dermyshi et al., 2017). The network meta-analysis by Wang et al. (2023) which included 106 trials involving 25,840 preterm infants, found that multiple-strain probiotics alone (risk ratio [RR] 0.38; 95% Confidence Interval [CI] 0.30 to 0.50) or in combination with oligosaccharides (RR 0.13; 95% CI 0.05 to 0.37) were among the most effective interventions for reducing severe NEC compared to placebo. Morgan et al. (2020) in another network meta-analysis of 63 trials involving 15,712 preterm infants, reported moderate- to high-quality evidence that combinations of *Lactobacillus* spp. and *Bifidobacterium* spp. (OR 0.35), *B. animalis subsp. lactis* (OR 0.31), *L. reuteri* (OR 0.55), and *L. rhamnosus* (OR 0.44) significantly reduced severe NEC. The meta-analysis by Dermyshi et al. (2017) which included both RCTs and observational studies, found that probiotics were associated with a reduction in severe NEC rates in VLBW infants, with RR of 0.57 (95% CI 0.47–0.70) in RCTs and 0.51 (95% CI 0.37–0.70) in observational studies.

Aceti et al. (2015) in an earlier meta-analysis, found that probiotics prevented NEC with a RR of 0.47 (95% CI 0.36–0.60), with strain-specific analyses showing significant effects for *Bifidobacteria* alone (RR 0.24) and for probiotic mixtures (RR 0.39). The review by Thye et al. (2026) further supported that probiotic supplementation in preterm infants has the potential to decrease NEC incidence, although inconsistent results due to strain differences and clinical heterogeneity limit widespread adoption.

Single-center observational studies have also provided supporting evidence. Robertson et al. (2020) reported that the introduction of routine multistrain probiotics was associated with a significant decrease in NEC rates from 7.5 to 3.1% (adjusted sub-hazard ratio 0.44, 95% CI 0.23 to 0.85) in a level 3 NICU over a 10-year period. Cases of LOS also fell from 22.6 to 11.5%, and there was no episode of sepsis due to *Lactobacillus* or *Bifidobacterium* (Robertson et al., 2020). The study by van Best et al. (2020) also reported a significantly reduced monthly incidence of NEC in neonates supplemented with probiotics. In that study, the P1 group received *Infloran* (250 mg/day; total 2 × 10^9^ CFU/day) and the P2 group received *DarmfloraPlus* (250 mg/day; total 4 × 10^9^ CFU/day), both continued until 36 weeks’ postmenstrual age.

However, it is important to acknowledge that not all studies have reported a significant benefit. Juber et al. (2021) in a single-center retrospective review at the University of Iowa, found no significant difference in rates of modified Bell’s stage ≥2a NEC in VLBW infants following routine administration of a multispecies probiotic (pre-probiotic 2.1% vs. post-probiotic 1.5%). All-cause mortality rates were also similar (pre-probiotic 8.4% vs. post-probiotic 7.4%). When restricted to ELBW infants, probiotic use had no impact on NEC incidence (pre-probiotics 1.6% vs. post-probiotics 4.1%) (Juber et al., 2021). Similarly, Priyadarshi et al. (2021) found no differences in NEC rates between infants receiving single-strain versus two-strain probiotic prophylaxis, although the study was not sufficiently powered to compare products. These discordant findings underscore the importance of contextual factors, including baseline NEC rates, population characteristics, and probiotic product specifics, in interpreting study results.

Subgroup analyses suggest that the clinical efficacy of probiotics may differ by GA. A large meta-analysis found that multi-strain probiotics were particularly effective in reducing NEC and related mortality in preterm infants aged 32–37 weeks and those with birth weight 1,500–2,500 g. Conversely, in a large implementation study, probiotic implementation was associated with a 51% lower risk of NEC after adjusting for GA and other confounders, and similar associations were observed among extremely preterm infants (ARR 0.49; 95% CI 0.34–0.72; *p* < 0.001) (Imren et al., 2026). The ProPrems trial subgroup analyses suggested efficacy in reducing NEC and sepsis in infants >1 kg and >28 weeks’ gestation, but not in smaller infants. These data underscore the need for GA-stratified analyses in future trials (Table 5).

**Table 5:** Clinical outcomes associated with probiotic supplementation in preterm infants.

Outcome	Effect estimate	Quality of evidence	Key references
NEC (Bell stage ≥2)	RR 0.38–0.57 (multi-strain); OR 0.31–0.55 (specific strains)	Moderate to high	Wang et al. (2023), Aceti et al. (2015), Morgan et al. (2020), and Dermyshi et al. (2017)
All-cause mortality	RR 0.69 (multi-strain); OR 0.56 (*Lactobacillus* + *Bifidobacterium*)	Moderate to high	Wang et al. (2023), Morgan et al. (2020) and Dermyshi et al. (2017)
Late-onset sepsis	RR 0.33 (with lactoferrin); inconsistent for probiotics alone	Low to moderate	Wang et al. (2023), Thye et al. (2026), Chang et al. (2022), Morgan et al. (2020), and Dermyshi et al. (2017)
Feeding intolerance	RR 0.45–0.61	Moderate	Wang et al. (2023), Qiao et al. (2017), Wang H. et al. (2025), and Ye et al. (2026)
Time to full enteral feeding	MD -1.94 to −3.30 days	Moderate to high	Wang et al. (2023) and Morgan et al. (2020)
Duration of hospitalization	MD -2.20 to −13.00 days (strain-dependent)	Moderate	Wang et al. (2023) and Morgan et al. (2020)
NEC surgery requirement	Reduced (limited data)	Low	Li and Peng (2026)
Neurodevelopmental outcomes	No significant difference	Low to very low	Abrahamsson et al. (2026) and Upadhyay et al. (2020)

MD, mean difference; NEC, necrotizing enterocolitis; OR, odds ratio; RR, risk ratio.

### 4.2 LOS

The evidence for probiotic efficacy in preventing LOS is more mixed than for NEC. Wang et al. (2023) in their network meta-analysis, found that single-strain probiotics in combination with lactoferrin were the most effective intervention for reducing sepsis (RR 0.33; 95% CI 0.14 to 0.78), whereas the effect of probiotics alone was less robust. Morgan et al. (2020) did not find moderate- or high-quality evidence for a significant reduction in culture-proven sepsis with any probiotic formulation compared to placebo. Dermyshi et al. (2017) reported a 12% reduction in the risk of sepsis in RCTs and a 19% reduction in observational studies.

Chang et al. (2022) in a study of extremely preterm infants, found that the probiotic group had a significantly lower rate of LOS compared to the control group (47.1% versus 70.0%, *p* = 0.015), although the rates of NEC and mortality were comparable. The probiotic group also had significantly fewer days of total parenteral nutrition (median 29.0 vs. 35.5, *p* = 0.004) (Chang et al., 2022). The PRIMAL trial, using a multi-strain formulation (*B. animalis* subsp. *lactis BB-12, B. longum* subsp. *infantis*, and *L. acidophilus La-5*; 1.5 × 10^9^ CFU of each strain per day for 28 days), found no significant difference in the incidence of LOS between the probiotic and control groups (2.5% vs. 3.7%) (Van Rossum et al., 2024). Similarly, the study by Athalye-Jape et al. (2025) comparing live versus heat-inactivated probiotics reported no cases of probiotic sepsis and found no significant difference in fecal calprotectin levels between groups.

The systematic review protocol by Iqbal et al. (2022) aims to evaluate the effect of probiotics in preterm neonates with sepsis, with the goal of translating evidence-based information to encourage the implementation of potential research output in the field of neonatal intensive care. The review by Thye et al. (2026) noted that probiotic supplementation in preterm infants has the potential to decrease NEC incidence and, to a lesser extent, sepsis/LOS, but inconsistent results due to strain differences and clinical heterogeneity limit widespread adoption (Table 5).

### 4.3 All-cause mortality

Probiotic supplementation has been associated with a reduction in all-cause mortality in preterm infants, although the evidence quality varies. Wang et al. (2023) reported that only multiple-strain probiotics were associated with reduced all-cause mortality compared to placebo (RR 0.69; 95% CI 0.56 to 0.86; risk difference −1.7%; 95% CI -2.4% to −0.8%). Morgan et al. (2020) found moderate- or high-quality evidence that combinations of *Lactobacillus* spp. and *Bifidobacterium* spp. reduced all-cause mortality (OR 0.56; 95% CI 0.39–0.80). Dermyshi et al. (2017) reported reductions in all-cause mortality in both RCTs (RR 0.77; 95% CI 0.65–0.92) and observational studies (RR 0.71; 95% CI 0.62–0.81).

Robertson et al. (2020) observed a decrease in all-cause mortality in the routine probiotics epoch from 14.3 to 9.2%, although this was not statistically significant after multivariable adjustment (adjusted sub-hazard ratio 0.74, 95% CI 0.49 to 1.12, *p* = 0.155). He et al. (2018) in a systematic review and meta-analysis of lactoferrin for preventing LOS and NEC, found that lactoferrin did not have a significant effect on all-cause mortality (RR 0.70; 95% CI 0.38–1.30). The review by Aceti et al. (2015) did not specifically report on all-cause mortality but noted that probiotics had an overall preventive effect on NEC (Table 5).

### 4.4 Feeding intolerance and growth outcomes

Feeding intolerance is a common complication in preterm infants that can delay the attainment of full enteral feeding and prolong hospitalization. Several studies have reported that probiotic supplementation is associated with a reduced incidence of feeding intolerance. Wang et al. (2023) found that multiple-strain probiotics alone (RR 0.61; 95% CI 0.46 to 0.80) or in combination with oligosaccharides (RR 0.45; 95% CI 0.29 to 0.67), as well as single-strain probiotics (RR 0.61; 95% CI 0.51 to 0.72), were among the most effective interventions for reducing feeding intolerance compared to placebo. Qiao et al. (2017) in a RCT of 60 preterm infants, found that probiotic supplementation with *Lactobacillus* and *Bifidobacterium* was associated with a lower rate of feeding intolerance (13.3% vs. 46.7%, *p* = 0.013). Wang H. et al. (2025) reported that *Bifidobacterium animalis* subsp. *lactis BB-12* supplementation at 1 × 10^9^ CFU/day (6 drops/day) was associated with a lower incidence of feeding intolerance, which was linked to increased levels of gut amino acids and reduced serum inflammatory markers. Ye et al. (2026) also found that early supplementation with *Bacillus subtilis* and *Enterococcus faecium* (1.5 × 10^8^ CFU/day for 14 days) was associated with a significantly lower incidence of feeding intolerance (17.07% vs. 44.74%; adjusted OR = 0.156, 95% CI 0.050–0.491) in antibiotic-exposed preterm infants.

The network meta-analysis by Wang et al. (2023) also reported that single-strain probiotics (mean difference −1.94 days; 95% CI –2.96 to −0.92) and multistrain probiotics (mean difference −2.03 days; 95% CI –3.04 to −1.02) were the most effective in reducing the time to reach full enteral feeding compared to placebo. Morgan et al. (2020) found moderate- to high-quality evidence that combinations of *Lactobacillus* spp. and *Bifidobacterium* spp. and *Saccharomyces boulardii* reduced the number of days to reach full feeding (mean reduction of 3.30 days; 95% CI reduction of 5.91–0.69 days). Single-species products *B. animalis* subsp. *lactis* and *L. reuteri* significantly reduced duration of hospitalization (mean reduction of 13.00 days and 7.89 days, respectively) (Morgan et al., 2020).

However, not all studies have demonstrated improvements in growth. Chrzanowska-Liszewska et al. (2012) found no significant difference in weight gain upon discharge or length of hospital stay in preterm infants supplemented with *L. rhamnosus GG* compared to placebo. Juber et al. (2021) also found no significant difference in all-cause pre-discharge mortality or NEC rates following routine probiotic administration. The mechanisms underlying these improvements in feeding tolerance may involve not only changes in microbial composition and reduced intestinal inflammation but also direct effects on gastrointestinal motility (Tables 5, 6).

**Table 6:** GA-specific considerations in microbiome development, probiotic response, and clinical outcomes.

GA stratum	Microbiome characteristics	Probiotic colonization/response	Clinical outcomes
EP (<28 weeks)	Persistent facultative anaerobe dominance; delayed obligate anaerobe colonization; lower *B. breve*	Colonization may be delayed; probiotic improves microbiota maturity and stability	Probiotic associated with 51% lower NEC risk (ARR 0.49); limited data on optimal strain/dose
VP (28–31 weeks)	Develops toward *Bifidobacterium*-dominated community; GA-dependent signature	Bifidobacterial colonization occurs at >32.9 weeks GA	ProPrems: efficacy in >28 weeks and >1 kg
MP/LP (32–36 weeks)	Microbiome resembles term infants; *Megamonas* and *Prevotella* emerge	Colonization more predictable	Multi-strain probiotics effective in 32–37 weeks

GA, gestational age; EP, extremely preterm; VP, very preterm; MP/LP, moderate-to-late preterm; NEC, necrotizing enterocolitis; ARR, adjusted risk ratio.

## 5 Mechanisms of probiotics action

### 5.1 Direct microbial modulation

Probiotics exert their effects through multiple interconnected mechanisms. The most direct mechanism is through competitive exclusion of pathogenic bacteria. By occupying ecological niches and competing for nutrients, supplemented probiotic strains can limit the colonization and overgrowth of potential pathogens (Rowland et al., 2010; Panigrahi, 2014). The production of antimicrobial substances, including organic acids, bacteriocins, and hydrogen peroxide, further creates an environment that is hostile to pathogenic bacteria (Samara et al., 2022; Alcon-Giner et al., 2020). The metabolic activity of supplemented probiotics, particularly *Bifidobacterium* and *Lactobacillus*, mainly yields acetate and lactate, which contribute to a lower intestinal pH. Propionate and butyrate are produced primarily by other commensal taxa or through cross-feeding interactions, rather than directly by the supplemented *Bifidobacterium* and *Lactobacillus* strains (Thatrimontrichai et al., 2025). Thye et al. (2026) in a recent review, highlighted that *Bifidobacterium* spp. and *Lactobacillus* spp. are the most commonly studied probiotic genera for NEC and sepsis prevention, with their effects mediated through both direct microbial competition and host immune modulation. The study by Alcon-Giner et al. (2020) demonstrated that fecal acetate positively correlated with relative abundance of *Bifidobacterium*, consistent with the ability of the supplemented *Bifidobacterium* strain to metabolize HMOs into acetate.

Panigrahi (2014), in a review on probiotics and prebiotics in neonatal NEC, discussed the uniqueness and differences among probiotic strains, and how multiple resident flora interact in the community setting in the human gut. The review highlighted the importance of understanding the developing microbiota in the neonatal gut and its immunological impacts on the host for selecting optimal probiotic candidates (Panigrahi, 2014). Rowland et al. (2010) in a report of an expert meeting, noted that probiotic effects were species and often strain specific, and that some probiotics were effective in reducing the incidence and duration of rotavirus diarrhea in infants, antibiotic-associated diarrhea in adults, and NEC in premature infants. In addition to direct competition and metabolite production, probiotic surface molecules—including lipoproteins, S-layer proteins, and unmethylated CpG-DNA—can interact with host pattern recognition receptors (PRRs) such as TLR2 and TLR9, initiating immunomodulatory signaling cascades (Lebeer et al., 2010; Rachmilewitz et al., 2004).

Recent work from the Besner laboratory has advanced probiotic delivery strategies to overcome the poor persistence of conventionally administered probiotics after enteral administration. Their studies demonstrated that *Limosilactobacillus reuteri* (Lr) delivered in its biofilm state on dextranomer microspheres (DM) loaded with maltose or sucrose significantly reduced histologic injury, improved host survival, decreased intestinal permeability, reduced intestinal inflammation, and altered the gut microbiome in animal models of NEC (Olson et al., 2018). These effects were abolished when DM or the gtfW gene (encoding an enzyme central to biofilm production) was absent from the Lr inoculum. The biofilm formulation also demonstrated superior performance in a piglet model of NEC, protecting both the intestines and the brain (Wala et al., 2023). This tunable delivery system provides persistent probiotic benefits from just a single dose, representing a promising next-generation approach to NEC prevention (Olson et al., 2018) (Table 7).

**Table 7:** Proposed mechanisms of action of probiotics in preterm infants.

Mechanism category	Specific mechanism	Evidence level	Key references
Direct microbial modulation	Competitive exclusion of pathogens	High	Rowland et al. (2010) and Panigrahi (2014)
	Production of antimicrobial substances (organic acids, bacteriocins)	Moderate	Samara et al. (2022) and Alcon-Giner et al. (2020)
	Fermentation mainly to acetate and lactate; propionate/butyrate produced by other taxa or via cross-feeding	Moderate	Alcon-Giner et al. (2020), Athalye-Jape et al. (2025), and Thatrimontrichai et al. (2025)
	Lowering of intestinal pH	Moderate	Alcon-Giner et al. (2020)
Immune modulation	Modulation of innate immune cell populations	Moderate	Smout et al. (2026), Rühle et al. (2024), and Qazi et al. (2024)
	Reduced TLR4/NF-κB signaling	Moderate	Wang H. et al. (2025)
	Decreased pro-inflammatory cytokines (IL-1β, TNF-α)	Moderate	Wang H. et al. (2025)
	Reduced intestinal inflammation (calprotectin)	Moderate	Larke et al. (2022) and Athalye-Jape et al. (2025)
	Altered T cell development (animal models)	Preliminary	Rühle et al. (2024)
Gut-brain axis	Modulation of GABAergic neurons	Preliminary	Chen et al. (2026)
	Improved spatial learning and reduced anxiety (animal models)	Preliminary	Qin et al. (2026)
	Increased butyric acid in CSF	Preliminary	Chen et al. (2026)
Gut-lung axis	Restoration of gut microbiota in RDS	Preliminary	Fu Y. et al. (2025) and Seo et al. (2026)
	Reduced systemic inflammation in BPD	Preliminary	Zhang et al. (2022)
Metabolic effects	Increased amino acid levels (glutamine, glutamic acid)	Moderate	Wang H. et al. (2025)
	Increased kynurenine	Preliminary	Wang H. et al. (2025)
	AhR activation by *L. reuteri*-derived tryptophan metabolites (biofilm state)	Preclinical	Sajankila et al. (2026)
Host receptor signaling	Probiotic surface molecules (lipoproteins, CpG-DNA) interacting with TLR2/TLR9	Preclinical/Moderate	Lebeer et al. (2010) and Rachmilewitz et al. (2004)
	PRR/GPCR/AhR-mediated microbial sensing in intestinal epithelial cells and immune cells	Mechanistic framework	Wan et al. (2023)
Gut motility	Increased gastric emptying; reduced fasting antral area; modulation of SCFAs, GLP-1, PYY, 5-HT; AhR-TRPA1 signaling on enteric neurons; TLR2-mediated ENS maintenance	Clinical (gastric emptying); Preclinical (mechanistic pathways)	Indrio et al. (2008), Zheng et al. (2022), Zhang et al. (2025), and Jiang et al. (2026)
Novel delivery	Biofilm-based *L. reuteri* delivery on dextranomer microspheres; enhanced persistence, reduced NEC	Preclinical	Olson et al. (2018) and Wala et al. (2023)

BPD, bronchopulmonary dysplasia; CSF, cerebrospinal fluid; RDS, respiratory distress syndrome; SCFAs, short-chain fatty acids; TLR4, Toll-like receptor 4.

### 5.2 Host receptor and metabolite signaling

Beyond direct microbial competition and metabolite production, probiotics influence intestinal biology through specific interactions with host receptors and signaling pathways. The first step in host–microbe interaction is the sensing of microbial components and metabolites by the host immune system, which is mediated by PRRs, G protein-coupled receptors (GPCRs), the aryl hydrocarbon receptor (AhR), and nuclear receptors expressed in intestinal epithelial cells and intestine-resident immune cells (Wan et al., 2023). Probiotic surface molecules, including lipoproteins, S-layer proteins, and CpG-DNA, can interact with PRRs such as TLR2 and TLR9, initiating signaling cascades that modulate both pro- and anti-inflammatory cytokine production (Lebeer et al., 2010; Rachmilewitz et al., 2004).

Among these pathways, AhR signaling has emerged as a particularly relevant mechanism in NEC pathogenesis. Decreased AhR signaling pathway activation is implicated in NEC development, and *Limosilactobacillus reuteri* (*L. reuteri*) catabolizes tryptophan into AhR ligands (Sajankila et al., 2026). Recent work from the Besner laboratory has demonstrated that delivery of *L. reuteri* in its biofilm state increases intestinal AhR ligand concentrations and enhances AhR activation, which suppresses experimental NEC in rodent models (Sajankila et al., 2026). In this model, *L. reuteri*-mediated AhR activation reduced intestinal injury and improved protection against NEC, likely through enhanced colonization when the probiotic is delivered in its biofilm state (Sajankila et al., 2026). Related *in vitro*/animal model work has shown that *Bifidobacterium longum BX06*-derived indole-3-acetic acid alleviates NEC-induced intestinal injury by activating AhR through indole-3-acetic acid production, thereby modulating TLR4-mediated inflammatory responses (Zhang et al., 2026). These findings are consistent with the broader concept that microbial tryptophan metabolites act as AhR ligands that enhance intestinal epithelial barrier function and modulate immune responses (Agus et al., 2018; Vázquez-Gómez et al., 2023).

The interaction between probiotics and host PRRs is not limited to TLR4/NF-κB signaling. The review by Wan et al. (2023) provides a comprehensive framework for understanding how the host senses gut microbes through multiple receptor families [mechanistic framework], including PRRs, GPCRs, and AhR, and how disruption of microbial sensing contributes to intestinal disease. This framework helps explain why probiotic effects are not solely attributable to direct microbial competition but also involve active host receptor-mediated signaling. It should be emphasized, however, that much of the evidence for AhR and PRR-mediated probiotic effects comes from animal models and *in vitro* studies; clinical confirmation in preterm infants is still lacking (Table 7).

### 5.3 Immune modulation

The interaction between probiotics and the developing immune system is a critical aspect of their mechanism of action. Preterm infants have an immature and functionally distinct immune system, characterized by altered monocyte characteristics and reduced TLR4 expression (Rühle et al., 2024; Qazi et al., 2024). Probiotics have been shown to modulate immune cell composition and function. Rühle et al. (2024) using a perinatal mouse model, found that probiotic exposure had profound effects on immune cell composition in the intestine, liver, and lungs of newborn mice, with reductions in myeloid and B cells and induction of T cells. These effects were particularly relevant during transition phases of microbiome development. The study also found that probiotics modulated T cell development in the thymus of newborn mice, contributing to a better understanding of the interaction of probiotics with the developing immune system (Rühle et al., 2024).

Qazi et al. (2024) in a human observational longitudinal analysis of extremely preterm infants, reported that probiotic supplementation was associated with distinct monocyte phenotypic characteristics, without significantly influencing their responsiveness. The study found that extremely preterm infants had persistent alterations in their monocyte characteristics that were further aggravated in chorioamnionitis cases, showing a markedly reduced TLR4 expression and hampered lipopolysaccharide-stimulated cytokine responses 14 days after birth (Qazi et al., 2024). Sepsis during the first weeks of life strongly associated with increased pro-inflammatory and reduced IL-10 responses, and probiotic supplementation was associated with distinct monocyte phenotypic characteristics (Qazi et al., 2024).

Smout et al. (2026) using a microbiota-humanized mouse model with fecal samples from the PRIMAL trial, demonstrated that probiotic-driven shifts in human-derived microbial communities attenuated immune cell development in mice and altered early-life infection outcomes, underscoring the complex, context-dependent effects of probiotics on the neonatal microbiota-immune axis. At steady-state, 3-week-old pups colonized with probiotic-conditioned microbiota exhibited markedly reduced populations of innate immune cells, particularly in the colon, with subtler effects in the small intestine and spleen (Smout et al., 2026). Upon enteropathogenic *Escherichia coli* challenge, pups harboring probiotic-conditioned microbiota displayed reduced growth and impaired bacterial clearance, correlating with diminished numbers of key innate immune cell populations (Smout et al., 2026).

The anti-inflammatory properties of probiotics have also been demonstrated through reductions in fecal calprotectin and pro-inflammatory cytokines (Wang H. et al., 2025; Larke et al., 2022). Larke et al. (2022) found that fecal calprotectin was lower in infants receiving *B. infantis* relative to *L. reuteri* and was negatively associated with the microbial metabolite indole-3-lactate in a human RCT substudy. Wang H. et al. (2025) reported that *Bifidobacterium animalis* subsp. *lactis BB-12* supplementation was associated with reduced levels of serum inflammatory markers TLR4, NF-κB, IL-1β, and TNF-α in a human RCT study. Athalye-Jape et al. (2025) found no significant difference in fecal calprotectin levels between live and heat-inactivated probiotic groups after 3 weeks of supplementation with a three-strain *Bifidobacterium* mixture (total 3 × 10^9^ CFU/day), suggesting that both formulations may have comparable effects on intestinal inflammation (Table 7).

### 5.4 Gut-brain and gut-organ axes

Emerging evidence suggests that probiotics may influence outcomes beyond the gastrointestinal tract through gut-organ axes. The gut-brain axis, in particular, has garnered attention as a potential pathway linking early-life microbiome composition to neurodevelopmental outcomes (Tao et al., 2026; Abrahamsson et al., 2026). Abrahamsson et al. (2026) in the PROPEL study (human observational), evaluated whether early gut microbiota development and *L. reuteri* supplementation were associated with neurodevelopment at 2 years in extremely preterm infants and found that microbiota composition constellations and lower microbial diversity, but not single bacteria, were associated with impaired neurodevelopment. Microbial maturation over the first month was discriminative for motor development, with higher abundance of *E. coli* and *Enterococcus* relative to *Cutibacterium* associated with impairment (Abrahamsson et al., 2026). *L. reuteri* supplementation did not seem to affect neurodevelopment via the gut microbiome (Abrahamsson et al., 2026).

Qin et al. (2026) in a preterm mouse model, demonstrated that supplementation with *Clostridium butyricum* and *Bifidobacterium* enhanced microbial diversity, improved spatial learning, and alleviated anxiety-like behaviors, likely through reducing *Enterobacteriaceae* and promoting *Lactobacillaceae*. The preterm control group exhibited elevated levels of *Enterobacteriaceae*, *Ruminococcaceae*, *Streptococcaceae*, and *Gemellaceae*, while *Lactobacillaceae* levels were higher in full-term controls and the probiotic-supplemented group at both timepoints (Qin et al., 2026). Behavioral tests showed that probiotic-supplemented mice performed better than preterm controls in spatial memory tasks and displayed reduced anxiety-like behaviors (Qin et al., 2026).

Chen et al. (2026) reported that probiotics restored GABAergic neurons and attenuated postnatal seizures in a periventricular leukomalacia rat model, suggesting a potential role for probiotics in preventing epilepsy after brain injury. Periventricular leukomalacia rats exhibited microgliosis, reduced density of premyelinating oligodendrocytes, and decreased myelin expression, and at postnatal days 85–90, these rats showed a significantly higher frequency of electrographic seizures and longer total seizure duration (Chen et al., 2026). Treatment with probiotics restored GABAergic neurons and significantly reduced seizure frequency and duration, modulated gut microbiota composition, increased levels of butyric acid in both fecal samples and cerebrospinal fluid (CSF), reduced cortical microglial activation, and lowered CSF levels of the proinflammatory cytokine TNF-α (Chen et al., 2026). Pärtty et al. (2013) in a randomized trial of preterm infants, found that early prebiotic and probiotic supplementation reduced fussing and crying, and the placebo group had a higher percentage of *Clostridium histolyticum* group bacteria in their stools than did the probiotic group (13.9% vs. 8.9%). Luoto et al. (2023) reported that probiotic and prebiotic treatments mitigated the shift in the microbiome of preterm infants by accelerating *Bifidobacteria*-dominated gut microbiome.

The gut-lung axis has also been implicated in preterm infant health. Zhang et al. (2022) found that BPD was associated with gut microbial alterations and elevated pro-inflammatory cytokines in a human observational study, suggesting that gut microbiota could influence BPD through inflammatory responses. Fu Y. et al. (2025) reported a human observational study that probiotic intervention in neonatal RDS helped restore disrupted gut microbiota and promoted disease recovery. Seo et al. (2026) in a translational study of neonatal RDS, established proof-of-concept for a disease-specific probiotic approach, demonstrating that candidate probiotic strains could increase microbial diversity and suppress opportunistic pathogens in an intestinal microbiota model. The study identified *Limosilactobacillus fermentum SLAM_LAF05* and *Bifidobacterium longum SLAM_BIL02* as candidate probiotic strains that were enriched in the control group and selected based on superior acid and bile tolerance and adhesion capacity (Seo et al., 2026). Additionally, the impact of non-invasive positive pressure ventilation on gut microbiota, as reported by Hutchinson et al. (2024) suggests that respiratory support itself may influence the intestinal environment, further highlighting the interconnectedness of these organ systems.

The systematic review by Upadhyay et al. (2020) synthesized literature on the effect of prebiotic and/or probiotic supplementation in preterm VLBW infants on neurodevelopmental outcomes and found that interventions did not decrease or increase the risk of cognitive and motor impairment, cerebral palsy, visual, and hearing impairment, with the quality of evidence being “low” to “very low.” The limited evidence from RCTs does not demonstrate a difference in neurodevelopmental outcomes between prebiotic/probiotic treated and untreated control groups (Upadhyay et al., 2020) (Table 7).

### 5.5 Effects on gut motility

Gastrointestinal dysmotility is a major clinical challenge in preterm infants and contributes substantially to feeding intolerance and delayed advancement of enteral nutrition. Several studies have investigated whether probiotic supplementation can directly improve gut motility in this population.

In a double-blind RCT of 30 preterm newborns, Indrio et al. (2008) showed that supplementation with *Lactobacillus reuteri ATCC 55730* (1 × 10^8^ CFU/day for 30 days) significantly increased the gastric emptying rate and reduced the fasting antral area compared with placebo, with reduced regurgitation and increased stool frequency. A subsequent RCT by Indrio et al. (2017) in formula-fed preterm newborns confirmed that *L. reuteri DSM 17938* (10^8^ CFU/day for one month) significantly shortened the time to full enteral feeding and improved gastric emptying, alongside a shift toward an anti-inflammatory cytokine profile. These clinical findings support the concept that probiotics may improve feeding tolerance partly through enhanced gut motility.

Four interconnected pathways may mediate probiotic effects on gut motility: (1) Microbial metabolites. SCFAs produced by microbial fermentation stimulate GLP-1 and PYY production and modulate 5-HT release, with dual effects on motility depending on concentration and context (Zheng et al., 2022). Tryptophan metabolites, particularly indole compounds, activate the AhR on enteric neurons; experimental evidence demonstrates that AhR activation restores intestinal smooth muscle strength and peristalsis frequency via the AHR-TRPA1 axis, reducing the inhibitory neurotransmitter NO production (Zhang et al., 2025). This mechanism is preclinical (zebrafish and rodent models) and requires confirmation in human preterm infants. (2) Enteroendocrine signaling. Enteroendocrine cells sense microbial signals and produce IL-22, which regulates early-life gut motility before immune system maturation (preclinical, zebrafish model) (Rabahi et al., 2026). Bacterial cell wall components—lipopeptides and peptidoglycan—signal through TLR2 expressed on enteric neurons and smooth muscle cells, maintaining the enteric nervous system and nitrergic neurons (Zheng et al., 2022). (3) Enteric nervous system. Probiotic-derived metabolites support neuronal survival and regeneration, and microbial signals regulate enteric neural pathways that control peristalsis (Jiang et al., 2026). Supplementation with SCFAs improves neuronal survival and regeneration, thereby ameliorating antibiotic-induced enteric nervous system injury (preclinical) (Vicentini et al., 2021). (4) Inflammation. Probiotics reduce TLR4/NF-κB-mediated inflammatory signaling in the immature gut, which may indirectly improve motility by reducing inflammatory inhibition of enteric neural function.

The observed reductions in feeding intolerance and time to full enteral feeding in probiotic trials may therefore be partly explained by improved gut motility, in addition to changes in microbial composition and reduced intestinal inflammation. However, direct human evidence for probiotic effects on motility in preterm infants remains limited, and most mechanistic data are derived from animal models. Future studies should include objective measures of gastrointestinal motility (e.g., gastric emptying, antral area, manometry) as outcomes in probiotic trials (Table 7).

## 6 Safety and challenges

### 6.1 Biological safety: probiotic-associated bacteremia and fungemia

The safety of probiotic administration in preterm infants, particularly those with ELBW, remains a subject of ongoing scrutiny. While most trials have reported no serious adverse events directly attributable to probiotics, case reports of probiotic-associated sepsis, although rare, have raised concerns (Juber et al., 2021; Thye et al., 2026). In the studies reviewed, no episodes of culture-proven sepsis with the probiotic organism were reported in the PRIMAL trial (which used *B. animalis* subsp. *lactis BB-12, B. longum* subsp. *infantis*, and *L. acidophilus La-5* at 1.5 × 10^9^ CFU of each strain per day for 28 days) or the study by Van Rossum et al. (2024) and Robertson et al. (2020). Athalye-Jape et al. (2025) found no cases of probiotic sepsis in their comparison of live versus heat-inactivated probiotics. The systematic review by Aceti et al. (2015) also did not report significant adverse effects related to probiotic supplementation. Moreno-Sanz et al. (2022) reported that none of the adverse events registered were related to product intake. Ye et al. (2026) reported no treatment-related adverse events in their study of *Bacillus subtilis* and *Enterococcus faecium* supplementation (1.5 × 10^8^ CFU/day for 14 days).

In September 2023, the U.S. FDA issued a warning following the death of a preterm infant weighing <1,000 g from probiotic-associated sepsis after receiving Evivo with MCT Oil. The FDA cautioned that microorganisms in probiotics have been reported to cause bacteremia or fungemia in very preterm or VLBW infants (U.S. Food and Drug Administration (FDA), 2023). However, the absolute risk appears extremely low: a 2025 meta-analysis identified only 8 cases among 20,323 exposed infants (<0.04%) (Feldman et al., 2025), and a 10-year single-center study reported 12 cases among 2,109 infants (0.6%) with no deaths directly attributed to bacteremia (Abda et al., 2025). Risk–benefit calculations estimated that per case of probiotic sepsis, an additional 62 NEC cases, 42 deaths, and 92 clinical sepsis events would occur in unexposed infants (Feldman et al., 2025).

However, Lefèvre et al. (2024) in a review of probiotic selection for NEC prevention, noted discrepancies between international recommendations and variability in available infant specialties, highlighting the challenges in establishing safe and effective protocols at the local level. The review noted that discrepancies between different recommendations appeared, and the great variability of infant specialties available, as well as their status, did not allow for selection of a single product (Lefèvre et al., 2024). The report by Rowland et al. (2010) noted that while some probiotics were effective in reducing NEC in premature infants, several other clinical effects of probiotics were thought promising but inconsistent (Table 8).

**Table 8:** Major challenges and knowledge gaps in probiotic research for preterm infants.

Challenge/Gap	Description	Potential solutions	Key references
Optimal strain selection	No consensus on best strain, combination, dosage, or duration	Head-to-head trials; strain-specific meta-analyses; mandatory reporting of full strain identity, dose, viability, and product composition	Wang et al. (2023), Thye et al. (2026), Lefèvre et al. (2024), and Morgan et al. (2020)
Product quality control	Label claims often inaccurate; pill-to-pill and lot-to-lot variation	Stricter regulation; third-party verification	Lewis et al. (2016)
Regulatory differences	U.S. FDA warning (2023) vs. ESPGHAN conditional recommendation (2023); divergent clinical practice across countries	Harmonized international standards; regulatory-grade products; clear risk–benefit guidance	van den Akker et al. (2024) and Mitha et al. (2026a)
Biological safety	Rare probiotic-associated bacteremia/fungemia (<0.04%); greatest risk in ELBW infants	Local laboratory capacity to detect probiotic sepsis; risk–benefit assessment; surveillance	Feldman et al. (2025) and Abda et al. (2025)
Methodological heterogeneity	Variable study designs, populations, and outcomes	Standardized protocols; core outcome sets; minimum reporting standards for strain identity, dose, viability, and product composition	Wang et al. (2023), Thye et al. (2026), and Aceti et al. (2015)
Safety in ELBW infants	Limited data; rare probiotic sepsis cases	Adequately powered trials in ELBW populations	Juber et al. (2021), Thye et al. (2026), and Aceti et al. (2015)
Antimicrobial resistance	Potential for horizontal gene transfer	Surveillance; metagenomic monitoring	Bargheet et al. (2023), Fernandes et al. (2024), and Hagbø et al. (2020)
Long-term outcomes	Limited data on neurodevelopment, metabolism, immunity	Long-term follow-up studies	Abrahamsson et al. (2026), Upadhyay et al. (2020), and Zimmermann et al. (2025)
Mechanistic understanding	Incomplete understanding of gut-organ axes	Multi-omics approaches; animal models	Tao et al. (2026), Smout et al. (2026), Rühle et al. (2024), and Chen et al. (2026)
Personalized approaches	One-size-fits-all may not be optimal	Stratification by GA, feeding type, antibiotic exposure	Jia et al. (2022) and Chen et al. (2024)
Implementation in LMICs	Limited data from low-resource settings	Trials in LMIC populations	Ford et al. (2025)
Synergistic combinations	Limited data on synbiotics, postbiotics	Dedicated trials of combination products	Wang et al. (2023), Navarro-Tapia et al. (2020), and Hortensius et al. (2021)
Formulation and administration	Single- vs. multi-strain; liquid vs. powder; dose, timing, duration, storage, vehicle variability	Standardized reporting; product-specific stability data; head-to-head trials	van den Akker et al. (2020) and Schimmoeller et al. (2022)

ELBW, extremely low birth weight; GA, gestational age; LMICs, low- and middle-income countries.

### 6.2 Product and regulatory safety: quality control, strain identity, and manufacturing standards

A significant challenge in the field is the quality control of commercial probiotic products. Lewis et al. (2016) in an evaluation of 16 commercial probiotic products, found that the contents of many *bifidobacterial* products differed from the ingredient list, sometimes at a subspecies level, with only one product perfectly matching its label claims. Pill-to-pill and lot-to-lot variation were also observed (Lewis et al., 2016). This lack of quality control has direct implications for both clinical trials and clinical practice, as variability in product composition may contribute to inconsistent study findings and limit the generalizability of results (Lewis et al., 2016). The study noted that given the known differences between various *bifidobacterial* species and subspecies in metabolic capacity and colonization abilities, the prevalence of misidentified *bifidobacteria* in these products is cause for concern for those involved in clinical trials and consumers of probiotic products (Lewis et al., 2016).

The selection of probiotics for clinical use is further complicated by the wide variety of available products, differences in strain-specific effects, and the lack of clear guidance from international recommendations (Thye et al., 2026; Lefèvre et al., 2024; Aceti et al., 2015). Priyadarshi et al. (2021) emphasized the challenges in selecting the optimal probiotic for clinical use due to variations in strain-specific effects, differences in administration protocols, and synergistic interactions with combination strains. The review by Thye et al. (2026) noted that inconsistent results due to strain differences and clinical heterogeneity limit the widespread adoption of probiotic therapy.

Most commercial probiotic products are regulated as dietary supplements rather than medicinal products, and no probiotic product is currently FDA-approved as a drug or biological product for use in infants (U.S. Food and Drug Administration (FDA), 2023). This regulatory gap has direct implications for product quality. European Society for Paediatric Gastroenterology, Hepatology and Nutrition (ESPGHAN) has emphasized that only products manufactured according to current good manufacturing practices should be used, that strains should be devoid of transferable antibiotic resistance genes, and that local microbiologists should be able to routinely detect probiotic sepsis. These requirements are not consistently met by currently available commercial products (van den Akker et al., 2020) (Table 8).

### 6.3 International regulatory frameworks and clinical practice

The regulatory environment and clinical practice surrounding probiotic administration to preterm infants differ substantially across countries. In the United States, the 2021 AAP Committee on Fetus and Newborn clinical report stated that current evidence does not support the routine, universal administration of probiotics to preterm infants, particularly those with a birth weight <1,000 g, due to the lack of FDA-regulated pharmaceutical-grade products, conflicting safety and efficacy data, and potential for harm (U.S. Food and Drug Administration (FDA), 2023). The 2023 FDA warning further restricted probiotic use, and a survey of U.S. NICUs found that following the warning, almost all respondents had ceased administering prophylactic probiotics (Wala et al., 2024).

In contrast, the ESPGHAN issued a conditional recommendation in 2023 (low certainty of evidence) that clinicians may recommend eithe*r Lactobacillus rhamnosus GG ATCC 53103* at a dose of 1–6 × 10^9^ CFU or a combination of *Bifidobacterium infantis BB-02*, *Bifidobacterium lactis BB-12*, and *Streptococcus thermophilus TH-4* (dose: 3–3.5 × 10^8^ CFU of each strain) to reduce NEC rates (Preidis et al., 2026). The European Foundation for the Care of Newborn Infants and the National Health Service of England have also advocated for the use of certain probiotic strains for NEC prevention (Wala et al., 2024).

The Swedish experience provides a notable contrast to the U.S. situation. After the Swedish Neonatal Society issued a national recommendation in 2020 to provide probiotics to very preterm infants born at 28^+0^–31^+6^ weeks, probiotic use increased from 0 to 65% across 38 Swedish NICUs. Probiotic supplementation was associated with a significant reduction in the risk of death and/or NEC (adjusted relative risk [ARR], 0.28; 95% CI, 0.14–0.56), overall NEC (ARR, 0.25; 95% CI, 0.10–0.61), surgical NEC (ARR, 0.04; 95% CI, 0.01–0.21), and mortality (ARR, 0.16; 95% CI, 0.06–0.45), with no associated increase in sepsis (ARR, 1.22; 95% CI, 0.78–1.90) (Patel, 2026; Mitha et al., 2026b). In contrast, the cessation of probiotic use in U.S. NICUs following the 2023 FDA warning has been associated with a 60% increase in the incidence of NEC (Patel, 2026) (Table 8).

These divergent approaches reflect differing interpretations of the same evidence base, differing regulatory frameworks, and differing tolerances for uncertainty in a highly vulnerable population.

### 6.4 Formulation and administration considerations

Studies of probiotics in preterm infants vary substantially in formulation, dosing, and administration, and these differences may influence organism viability, delivered dose, colonization, safety, and efficacy. One key source of variation is the number of strains included in the product. Meta-analyses suggest that multi-strain probiotics are associated with a significant reduction in NEC (pooled OR 0.36) (Chang et al., 2017), whereas single-strain probiotics show only a borderline effect, although head-to-head comparisons remain limited (Morgan et al., 2020; van den Akker et al., 2020).

Formulation type also differs across studies, with powdered products typically requiring reconstitution and being susceptible to moisture and temperature fluctuations, while liquid formulations may have different viability profiles (Watkins et al., 2018; Schimmoeller et al., 2022). Daily doses in published trials range from 6 × 10^7^ CFU/day (Gong et al.) to more than 2 × 10^14^ CFU per sachet (ProPrems), with most studies using 1 × 10^9^ to 4 × 10^9^ CFU/day (Gong et al., 2021). The timing of initiation also varies, from within 24 h of birth to after the establishment of enteral feeding, and duration ranges from 7 days to continuation until 36 weeks postmenstrual age or discharge (Moreno-Sanz et al., 2022). Viability at the time of administration depends on storage conditions, reconstitution procedures, and the vehicle used, whether human milk, formula, or glucose solution (Watkins et al., 2018). Administration with human milk may enhance colonization of HMO-utilizing strains such as *B. infantis* (Underwood et al., 2015; Underwood et al., 2013).

Collectively, these differences may contribute to the heterogeneity in study findings and limit the generalizability of results. Future trials should report full strain identity, daily dose (CFU/day), viability at administration, product composition, and administration procedures to permit strain- and formulation-specific synthesis (Table 8).

### 6.5 Methodological heterogeneity

The variability in study designs, probiotic strains, dosages, administration protocols, and patient populations across studies represents a major obstacle to synthesizing evidence and establishing standardized guidelines. Aceti et al. (2015) noted that the majority of studies included in their meta-analysis presented severe or moderate microbiological flaws, including inadequate characterization of the baseline microbiota and lack of strain-specific identification. The network meta-analysis by Wang et al. (2023) included 106 trials with substantial heterogeneity in probiotic products and outcomes, highlighting the difficulty in making direct comparisons. The review by Thye et al. (2026) noted that further high-quality standardized studies are necessary to establish consistent guidelines for probiotic use in preterm infants. A minimum reporting standard should include full strain identity, dose, viability at administration, and product composition.

The systematic review by Dermyshi et al. (2017) which included both RCTs and observational studies, found that while probiotics were beneficial for the prevention of severe NEC, LOS, and all-cause mortality in VLBW infants, the heterogeneity among studies necessitated careful interpretation of results. The review by Navarro-Tapia et al. (2020) addressed the importance of probiotics as a preventive or therapeutic tool for dysbiosis produced during the perinatal and infant period, and discussed the safety of the use of probiotics in pregnant women, preterm neonates, or infants for the treatment of atopic diseases and infections. The study protocol by Martí et al. (2021) provided an optimized protocol for fecal DNA isolation, library preparation for 16S rRNA gene sequencing, taxonomy assignation, and statistical analyses, which is customizable for other clinical trials and may help standardize methodologies across studies. In addition to strain heterogeneity, daily dose and duration of probiotic administration varied widely across studies or were not reported, further complicating meta-analytic synthesis (Table 8).

### 6.6 Antimicrobial resistance

The impact of probiotics on antimicrobial resistance is an emerging concern. Preterm infants frequently receive antibiotics, which can select for antibiotic-resistant bacteria in the gut (Bargheet et al., 2023; Gong et al., 2021). Probiotics themselves may carry antibiotic resistance genes, raising the possibility of horizontal gene transfer to pathogenic bacteria (Fernandes et al., 2024; Hagbø et al., 2020). Fernandes et al. (2024) in a systematic review, found that the impact of probiotics on antibiotic resistance in newborns varied across studies, with two studies suggesting a positive impact, one a negative impact, and three finding no significant impact. The review highlighted that microbiota changes were addressed across four studies, where the findings included promotion of beneficial bacteria and reduction of potential pathogens, but the observed heterogeneity between studies did not allow for clear conclusions (Fernandes et al., 2024).

Hagbø et al. (2020) experimentally demonstrated frequent transmission of an *ESBL-encoding IncI1* plasmid, a high copy number *ColEI* plasmid, and *bacteriophage P1* in the preterm infant gut microbiota, supporting a major horizontal transmission potential of the mobilome involving genes encoding extended-spectrum beta-lactamases. *In vitro* competition experiments showed that transconjugants containing *IncI1* plasmids outcompeted the recipient strain in the absence of antibiotic selection, and the Sequence Read Archive searches indicated a global distribution of the mobilizable elements, with chicken identified as a possible reservoir for the *IncI1 ESBL* encoding plasmid (Hagbø et al., 2020).

Bargheet et al. (2023) reported that gestational age, hospitalization, and both microbiota-modifying treatments (antibiotics and probiotics) contributed to elevated carriage of mobile genetic elements in preterm infants compared to term controls. The top predictors of microbiota maturation were hospitalization length and gestational age, and probiotic administration rendered the gut microbiota and resistome of extremely preterm infants more alike to term infants on day 7 and ameliorated GA-driven loss of microbiota interconnectivity and stability (Bargheet et al., 2023). *Escherichia coli* was associated with the highest number of antibiotic-resistance genes, followed by *Klebsiella pneumoniae* and *Klebsiella aerogenes* (Bargheet et al., 2023). Mazandarani et al. (2026) in a systematic review, found that incorporating probiotics and synbiotics into common infection therapy could improve antibiotic resistance, eradicate rates, and minimize side effects, but concerns remain regarding the potential transfer of antibiotic resistance genes via probiotics (Table 8).

## 7 Knowledge gaps and future directions

Despite the substantial body of evidence supporting the use of probiotics in preterm infants, several critical knowledge gaps remain. These gaps are organized below by theme to facilitate navigation and to highlight priority areas for future research.

### 7.1 Optimal strain, dose, and duration

The optimal probiotic strain, combination, dosage, and duration of administration have not been definitively established (Wang et al., 2023; Morgan et al., 2020). Because probiotic effects are strain-specific, evidence for one formulation cannot be extrapolated to other strains or products. The variability in study findings suggests that strain-specific effects are important, and head-to-head comparisons of different products are needed. Future trials must report full strain identity, dose, viability, and product composition to permit strain- and formulation-specific synthesis. The network meta-analyses by Wang et al. (2023) and Morgan et al. (2020) provide some guidance, but the heterogeneity among studies limits the generalizability of findings. The review by Thye et al. (2026) emphasized the need for further high-quality standardized studies to establish consistent guidelines for probiotic use in preterm infants.

### 7.2 Mechanistic understanding and multi-omics approaches

The mechanisms by which probiotics exert their clinical effects are incompletely understood, particularly with respect to the gut-brain, gut-lung, and other gut-organ axes (Tao et al., 2026; Abrahamsson et al., 2026; Seo et al., 2026). Advanced multi-omics approaches, including metagenomics, metabolomics, and metatranscriptomics, combined with machine learning and digital twin modeling, are beginning to provide more detailed insights into host–microbe interactions and may pave the way for personalized, predictive microbiome-mediated care (Tao et al., 2026). The review by Tao et al. (2026) highlighted that embracing the complexity of the preterm microbiome as a dynamic, multi-kingdom ecosystem is essential for developing holistic interventions that improve both short-term survival and long-term neurodevelopmental, respiratory, and metabolic outcomes.

### 7.3 Safety in extremely low-birth-weight infants

The safety of probiotics in the most vulnerable populations, particularly ELBW infants, requires further investigation through adequately powered trials (Juber et al., 2021; Aceti et al., 2015). The review by Aceti et al. (2015) noted that there were insufficient data for extremely-low-birth-weight infants before a widespread use of these products can be recommended. The study by Juber et al. (2021) found that probiotic use had no impact on NEC incidence in ELBW infants, highlighting the need for more research in this population. Athalye-Jape et al. (2025) compared live versus heat-inactivated probiotics and suggested that heat-inactivated probiotics may provide an effective alternative to live probiotics by avoiding risks such as probiotic sepsis while retaining benefits, but adequately powered trials are needed to assess the effects on clinically significant outcomes.

### 7.4 Long-term neurodevelopmental and metabolic outcomes

The long-term consequences of early probiotic exposure, including effects on neurodevelopment, metabolic health, and the immune system, remain to be fully elucidated (Abrahamsson et al., 2026; Upadhyay et al., 2020). The systematic review by Upadhyay et al. (2020) which found limited evidence from RCTs demonstrating a difference in neurodevelopmental outcomes between probiotic-treated and untreated groups, highlights the need for long-term follow-up studies. The NapBiome trial, currently underway, aims to investigate whether synbiotic administration improves sleep patterns and neurodevelopmental outcomes in preterm infants, representing a step toward more holistic interventions targeting the gut-brain axis (Zimmermann et al., 2025). The NutriBrain study protocol by Hortensius et al. (2021) aims to evaluate the effects of a combination of probiotics, prebiotics and L-glutamine on brain development in preterm infants.

### 7.5 Antimicrobial resistance and horizontal gene transfer

The impact of probiotics on antimicrobial resistance in the preterm gut requires ongoing surveillance, particularly given the potential for horizontal gene transfer (Bargheet et al., 2023; Fernandes et al., 2024). The studies by Bargheet et al. (2023) and Hagbø et al. (2020) have demonstrated the potential for horizontal transmission of antibiotic resistance genes in the preterm gut microbiota, emphasizing the need for careful monitoring. Fernandes et al. (2024) highlighted the need for further studies to clarify the impact of probiotics on antibiotic resistance in newborns.

### 7.6 Emerging strategies: heat-inactivated probiotics, postbiotics, phage therapy, and FMT

Emerging strategies beyond traditional live probiotics warrant exploration. Heat-inactivated probiotics, which may retain some beneficial effects while avoiding the risks associated with live organisms, have shown promising results in preliminary studies (Athalye-Jape et al., 2025). Postbiotics, phage therapy, and fecal microbiota transplantation (FMT) represent alternative or complementary approaches to microbiome modulation that may offer advantages in specific clinical contexts (Tao et al., 2026; Prado et al., 2022). The study by Prado et al. (2022) comparing fresh and sterile FMT in an experimental NEC model, found that both fresh and sterile FMT effectively reduced inflammation, oxidative damage, and histological alterations compared to an experimental NEC model. The review by Xiang et al. (2023) discussed different strategies of early intervention, such as probiotics, FMT, and nutrients, showing different safety, applicability, and outcome in premature infants, and noted that the underlying mechanism is complex and poorly understood. The protocol by Barreiros Mota et al. (2019) aims to explore the microbial colonization over time of the proximal intestinal remnant in newborn infants with enterostomy, which may serve as basis for future observational and interventional studies on the modulation of the intestinal microbiota on short and long-term outcomes in this population.

Biofilm-based probiotic delivery represents a particularly promising next-generation strategy. The Besner laboratory has developed a tunable delivery system in which *L. reuteri* administered in a protective biofilm on biocompatible microspheres provides persistent probiotic benefits from a single dose, greatly reducing experimental NEC (Olson et al., 2018). Their recent review discusses the challenges imposed on probiotic therapy by the 2023 FDA warning and examines novel delivery methods that can enhance the beneficial capabilities of probiotics (Sajankila et al., 2023). By obtaining approval for novel oral probiotic delivery technologies including biofilm formation and prebiotic co-delivery, the risk of NEC in premature infants could be significantly decreased. However, these approaches remain preclinical and require validation in human trials.

### 7.7 Synbiotics and combination products

The combination of probiotics with prebiotics (synbiotics) may enhance colonization and persistence of beneficial bacteria (Wang et al., 2023; Navarro-Tapia et al., 2020; Hortensius et al., 2021). The review by Navarro-Tapia et al. (2020) discussed the importance of probiotics as a preventive or therapeutic tool for dysbiosis produced during the perinatal and infant period. The study protocols by Hortensius et al. (2021) and the NapBiome trial represent ongoing efforts to evaluate synbiotic interventions in preterm infants (Zimmermann et al., 2025). The protocol by Wang N. et al. (2025) investigates the impact of oropharyngeal colostrum administration on postnatal growth in preterm infants based on early gut microbiota-host interaction patterns. The review by Chang et al. (2024) emphasized the need for further research to identify probiotic strains that exert significant influence on the gut microbiome and their long-term health implications in preterm infants.

### 7.8 Personalized microbiome modulation

The development of personalized approaches to microbiome modulation based on individual characteristics such as gestational age, birth weight, feeding type, and antibiotic exposure history may improve the efficacy of probiotic interventions (Jia et al., 2022; Chen et al., 2024). The study by Jia et al. (2022) profiled the features of dynamic changes of gut microbiome from different gestational ages infants, providing new insights for individualized interventions of specific microbes of preterm infants with different gestational ages at different time points after birth. The study by Chen et al. (2024) found that the gut bacteria of preterm infants showed community state types with *Enterococcus*, *Klebsiella*, and *Acinetobacter* as the dominant genera, and that ELBW preterm infants exhibit an increase in the abundance of potentially harmful bacteria and a decrease in beneficial bacteria, suggesting that these potentially harmful bacteria may be potential biomarkers for ELBW preterm infants. Stratification by gestational age, feeding type, and antibiotic exposure should be incorporated into future trial designs.

### 7.9 Implementation in low- and middle-income countries

The protocol by Fu Y. et al. (2025) and Ford et al. (2025) aims to assess the feasibility, safety, and initial outcomes of administering synbiotic formulations to newborns in Papua New Guinea, which may inform the design of larger trials of probiotics and synbiotics to complement existing efforts to reduce infection-related infant mortality in low- and middle-income countries. Given that the burden of preterm birth and NEC is highest in low-resource settings, implementation research addressing product stability, cold-chain requirements, affordability, and local regulatory pathways is urgently needed.

## 8 Conclusion

Probiotic supplementation represents a promising intervention for modulating gut microbiota and reducing morbidity in preterm infants, but the strength of evidence differs substantially across outcomes. The strongest evidence supports a reduction in severe NEC (Bell stage ≥2) with multi-strain formulations containing *Lactobacillus* and *Bifidobacterium* species, and a probable reduction in all-cause mortality with multi-strain products. Probiotics may also reduce feeding intolerance and time to full enteral feeding, with moderate certainty. In contrast, evidence for preventing LOS is inconsistent, and probiotics do not appear to reduce colonization with multidrug-resistant organisms. Neurodevelopmental outcomes show no demonstrated clinical benefit, with low to very low certainty, and evidence for BPD/RDS remains preliminary. Mechanistically, probiotics may act through direct microbial competition, production of antimicrobial metabolites, modulation of host immunity, and gut-organ axes including the gut-brain and gut-lung axes; however, many of these mechanisms are supported mainly by preclinical evidence and require confirmation in human preterm infants. Because probiotic effects are strain-specific and dose-dependent, findings should not be extrapolated across strains, species, or products. Routine universal probiotic administration cannot be recommended based on current evidence alone, especially in extremely low-birth-weight infants. Translation into standardized clinical practice requires product-specific, adequately powered trials, improved mechanistic understanding, and regulatory-grade quality control.
